# Complementary task representations in hippocampus and prefrontal cortex for generalizing the structure of problems

**DOI:** 10.1038/s41593-022-01149-8

**Published:** 2022-09-28

**Authors:** Veronika Samborska, James L. Butler, Mark E. Walton, Timothy E. J. Behrens, Thomas Akam

**Affiliations:** 1 Wellcome Centre for Integrative Neuroimaging, University of Oxford, Oxford, UK; 2 Department of Clinical and Movement Neurosciences, University College London, London, UK; 3 Sainsbury Wellcome Centre for Neural Circuits and Behaviour, University College London, London, UK; 4 Department of Experimental Psychology, University of Oxford, Oxford, UK; 5 Wellcome Centre for Human Neuroimaging, University College London, London, UK

**Keywords:** Intelligence, Problem solving

## Abstract

Humans and other animals effortlessly generalize prior knowledge to solve novel problems, by abstracting common structure and mapping it onto new sensorimotor specifics. To investigate how the brain achieves this, in this study, we trained mice on a series of reversal learning problems that shared the same structure but had different physical implementations. Performance improved across problems, indicating transfer of knowledge. Neurons in medial prefrontal cortex (mPFC) maintained similar representations across problems despite their different sensorimotor correlates, whereas hippocampal (dCA1) representations were more strongly influenced by the specifics of each problem. This was true for both representations of the events that comprised each trial and those that integrated choices and outcomes over multiple trials to guide an animal’s decisions. These data suggest that prefrontal cortex and hippocampus play complementary roles in generalization of knowledge: PFC abstracts the common structure among related problems, and hippocampus maps this structure onto the specifics of the current situation.

## Abstract (web summary)

Samborska et al. trained mice on a set of problems with the same structure but different physical layouts to study generalization. Neurons in prefrontal cortex generalized across problems, whereas those in hippocampus were more problem specific.

## Main

When we walk into a new restaurant, we know what to do. We might find a table and wait to be served. We know that the starter will come before the main, and when the bill arrives, we know it is the food we are paying for. This is possible because we already know a lot about how restaurants work and only have to map this knowledge onto the specifics of the new situation. This requires that the common structure is abstracted away from the sensorimotor specifics of experience, so it can be applied seamlessly to new but related situations.

Such abstraction has been variously described as a schema (in the context of human behavior^1^ and memory research^2,3^), learning set^4^ (in the context of animal reward-guided behavior), transfer learning^5^ and meta-learning^6^ (in the context of machine learning). We have little understanding of how the necessary abstraction is achieved in the brain or how abstract representations are tied to the sensorimotor specifics of each new situation. However, recent data suggest that interactions between frontal cortex and the hippocampal formation play an important role^7^. Neurons^8,9^ and fMRI voxels^10,11^ in these brain regions form representations that generalize over different sensorimotor examples of tasks with the same structure and track different task rules embedded in otherwise similar sensory experience^12,13^.

Both frontal cortex^14–17^ and hippocampus^18–27^ have been hypothesized to represent task states and the relationships between them. It has not been clear what distinguishes the representations in these regions, but insight might be gained by considering spatial cognition. In rodent hippocampus, place cells are specific to each particular environment^28–30^, but firing patterns in neighboring entorhinal cortex (including grid cells) generalize across different environments—that is, they are abstracted from sensorimotor particularities^31–35^. Similarly, there is evidence that mPFC representations of spatial tasks generalize across different paths^36–38^.

One possibility is that, as in space, abstracted or schematic representations of tasks in cortex are flexibly linked with the sensorimotor characteristics of a particular environment to rapidly construct concrete task representations in hippocampus, affording immediate inferences^39,40^. Indeed, hippocampal manipulations appear particularly disruptive when new task rules must be inferred, either early in training^41^ or when contingencies change^42,43^.

To probe cortical and hippocampal contributions to generalization, we developed a behavioral paradigm where mice encountered a series of problems with the same abstract structure (probabilistic reversal learning) but different physical instantiations and, hence, different sensorimotor correlates. We recorded single units in mPFC and hippocampus across multiple problems in each recording session. We examined neuronal representations of both the individual elements of each trial and the cross-trial learning that controlled animals’ choices. Both mPFC and dCA1 representations of trial events were low dimensional—that is, a small set of temporal patterns of activity, corresponding to tuning for particular trial events, explained a large fraction of variance in both regions. However, they differed with respect to how these representations generalized across problems. In mPFC, the same neurons tended to represent the same events across problems, irrespective of the sensorimotor particulars of the current problem. By contrast, although the same events were represented by hippocampus in each problem, the specific neurons that represented a given event differed in each problem. Both hippocampus and prefrontal cortex (PFC) also contained representations of animals’ current policy that integrated events over multiple trials. These policy representations were again abstract in PFC but tied to sensorimotor specifics in hippocampus.

## Results

### Mice generalize knowledge between problems

**Fig. 1:**
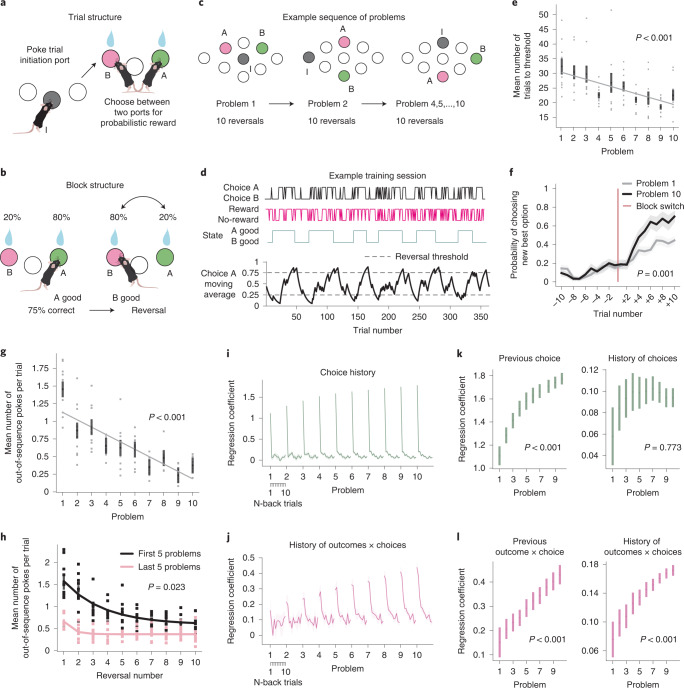
Transfer learning in mice. **a**, Trial structure of the probabilistic reversal learning problem. Mice poked in an initiation port (gray) and then chose between two choice ports (green and pink) for a probabilistic reward. **b**, Block structure of the probabilistic reversal learning problem. Reward contingencies reversed after the animal consistently chose the high reward probability port. **c**, Example sequence of problems used for training, showing different locations of the initiation (I) and two choice ports (A and B) in each problem. **d**, Example behavioral session late in training in which the animal completed 12 reversals. Top sub-panels show animals’ choices, outcomes they received and which side had high reward probability; bottom panel shows exponential moving average of subjects’ choices (tau = 8 trials). **e**, Mean number of trials after a reversal taken to reach the threshold to trigger the next reversal, as a function of problem number. **f**, Probability of choosing the new best option (the choice that becomes good after the reversal) on the last ten trials before the reversal and the first ten trials after the reversal split by the first problem and the last problem. The *P* value refers to the difference between the slopes after the reversal point in early and late training (paired-sample *t*-test, two-sided). **g**, Mean number of pokes per trial to a choice port that was no longer available because the subject had already chosen the other port, as a function of problem number. **h**, Mean number of pokes per trial to a choice port that was no longer available as a function of reversal number on the first five problems and the last five problems during training. The *P* value refers to the difference in the log of the time constants from fitted exponential curves in early and late training (paired-sample *t*-test, two-sided). **i**,**j**, Coefficients from a logistic regression predicting current choices using the history of previous choices (**i**), outcomes (not shown) and choice × outcome interactions (**j**). For each problem and predictor, the coefficients at lag 1–11 trials are plotted. **k**,**l**, Coefficients for the previous trial (lag 1, left) and average coefficients across lags 2–11 (right), as a function of problem number (*P* values derived from repeated-measures one-way ANOVAs with problem number as the within-subjects factor). Error bars on all plots show mean ± s.e.m. across mice (*n* = 9 mice). *P* values in **e** and **g** are from the two-way repeated-measures ANOVAs with problem number and reversal number as within-subjects factors.

Subjects serially performed a set of reversal learning problems that shared the same structure but had different physical layouts. In each problem, every trial started with an ‘initiation’ nose-poke port lighting up. Poking this port illuminated two ‘choice’ ports, which the subject chose between for a probabilistic reward (Fig. 1a). Once the subject consistently (75% of trials) chose the high reward probability port, reward contingencies reversed (Fig. 1b). Once subjects completed ten reversals on a given port layout (termed a ‘problem’), they were moved onto a new problem where the initiation and choice ports were in different physical locations (Fig. 1c). All problems, therefore, shared the same trial structure (initiate then choose) and a common abstract rule (one port has high and one has low reward probability, with reversals) but required different motor actions due to the different port locations. In this phase of the experiment, problem switches occurred between sessions, and subjects completed ten different problems.

**Extended Data Fig. 1:**
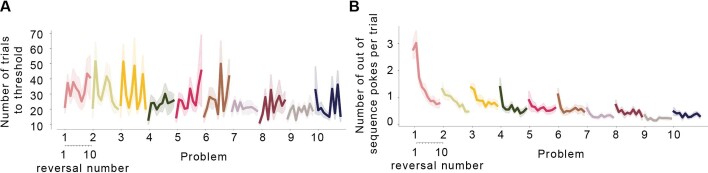
Transfer learning in mice. **a)** Number of trials following a reversal taken to reach the threshold to trigger the next reversal, as a function of reversal number within each problem and problem number. **b)** Number of pokes per trial to a choice port that was no longer available because the subject had already chosen the other port, as a function of reversal number within each problem and problem number. Shaded area indicates the mean ± SEM across mice (*n* = 9).

We first asked whether subjectsʼ performance improved across problems, consistent with their generalizing the problem structure (one port is good at a time, with reversals) (Fig. 1b). Mice took fewer trials to reach the 75% correct threshold for triggering a reversal within each problem (*F*_9,72_ = 3.52, *P* = 0.001; Extended Data Fig. 1a) and, crucially, also across problems (*F*_9,72_ = 3.91, *P* < 0.001; Fig. 1e), consistent with generalization. Improvement across problems tracking the good port might reflect an increased ability to integrate the history of outcomes and choices across trials. To assess this, we fit a logistic regression model predicting choices, using the recent history of choices, outcomes and choice × outcome interactions. Across problems, the influence of both the most recent (*F*_9,71_ = 5.08, *P* < 0.001; Fig. 1j,l) and earlier (*F*_9,71_ = 5.46, *P* < 0.001; Fig. 1j,l) choice × outcome interactions increased. Subjects’ choices were also increasingly strongly influenced by their previous choices (*F*_9,71_ = 11.77, *P* < 0.001; Fig. 1i,k), suggesting a decrease in spontaneous exploration.

**Extended Data Fig. 2:**
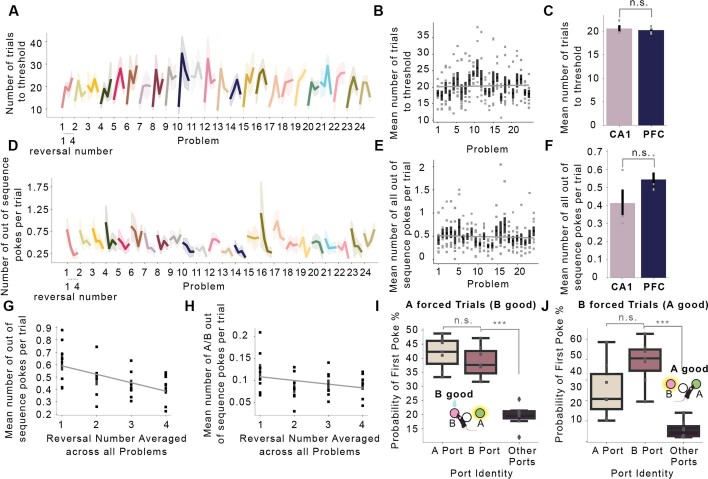
Behaviour during recordings. **a)** Number of trials following a reversal taken to reach the threshold to trigger the next reversal, as a function of reversal number within each problem (*F*_(3, 18)_ = 24.19, *p* < .001) and problem number (*F*_(23, 138)_ = 1.47, *p* = .09) during recordings. **b)** Average number of trials following a reversal taken to reach the threshold to trigger the next reversal, as a function of problem number during recordings (analogous to Fig. 1e). **c)** There was no significant difference in the mean number of trials animals took to reach the threshold for a reversal during recordings between PFC and CA1 animals (*t*_(7)_ = 0.42, *p* = .690). **d)** Number of pokes per trial to a choice port that was no longer available because the subject had already chosen the other port, as a function of reversal number within each problem (*F*_(3, 18)_ = 11.12, *p* < .001) and problem number (*F*_(23, 138)_ = 0.10, *p* = .474) during recordings. **e)** Average number of out of sequence pokes mice made as a function of problem number during recordings (analogous to Fig. 1g). **f)** There was no significant difference in the mean number of out of sequence pokes during recordings between PFC and CA1 animals (*t*_(7)_ = 1.39, *p* = .220). **g, h)** Mice made more out of sequence pokes per trial during the first reversal block compared to every following reversal (Reversal 1 vs Reversal 2: *t*_(167)_ = 2.61, *p* = .015), 1 vs 3: *t*_(167)_ = 3.84, *p* = .001; 1 vs 4: *t*_(167)_ = 4.15, *p* < .001). **g)** All out of sequence pokes plotted as a function reversal block number within problem. **h)** Out of sequence A/B choice pokes plotted as a function reversal block number within problem **i-j)** Mice did not just follow the lights to complete a trial. **I)** On forced A trials where A choice was illuminated but B choice was good animals were as likely to first choose the B choice port (not illuminated but good port at the time) as the illuminated A port (*t*_(7)_ = 0.76, *p* = .470) and were much more likely to choose the good B port than other ports (initiation and ports used in other problems) (*t*_(7)_ = 6.25, *p* <.001). **J)** On forced B trials where B choice was illuminated but A choice was good animals were as likely to first choose the A choice port as the B port (*t*_(7)_ = 1.45, *p* =.200), and were more likely to choose the A choice than the other ports (*t*_(7)_ = 2.94, *p* =.030). Error bars report the mean ± SEM (A-I) or median, inter quartile range and min and max(I-J) across mice (*n* = 7), dots show individual subjects.

We also asked whether subjects generalized the trial structure (initiate then choose; Fig. 1a) across problems, by assessing how often they made nose-pokes inconsistent with this sequence (that is, pokes to the alternative choice port after having made a choice, instead of returning to initiation). Mice made fewer out-of-sequences pokes across reversals within each problem (*F*_9,72_ = 17.82, *P* < 0.001; Extended Data Fig. 1b) but, notably, also across problems (*F*_9,72_ = 18.29, *P* < 0.001; Fig. 1g). This improvement was not just driven by animals’ poor performance on the first problem but continued throughout training (*F*_9,64_ = 9.36, *P* < 0.001). To assess whether it was driven simply by learning to follow port illumination, we examined behavior on ‘forced choice’ trials where only one choice port illuminated, and the other was inactive. Animals did not just follow the light and were equally likely to poke the high reward probability choice port as the choice port that was illuminated, demonstrating that their behavior was influenced by their belief about reward availability and not just the port illumination (Extended Data Fig. 2i,j), although it remains possible that they used port illumination while acquiring a new problem.

This observed improvement across problems is consistent with meta-learning (or ‘learning to learn’). In line with this, on early problems mice learned the new poke sequences necessary to execute trials gradually over many reversals, suggesting instrumental learning. However, at the end of the training, they acquired the new poke sequence in a single reversal, suggesting that they ‘learned how to learn’ the sequence (*t*_17_ = 2.81, *P* = 0.023; Fig. 1h). Similarly, animals adapted to reversals faster at the end of training compared to the beginning of training (*t*_17_ = 5.04, *P* = 0.001; Fig. 1f). Therefore, they had also ‘learned how to learn’ from reward.

These data demonstrate generalization but do not provide a mechanism. A possible mechanism is task abstraction, whereby the brain uses the same neuronal representation for different physical situations that play the same task role. To investigate whether such representations existed, we next examined cellular responses in mPFC and hippocampus.

### Abstract and problem-specific representations in PFC and CA1

**Fig. 2:**
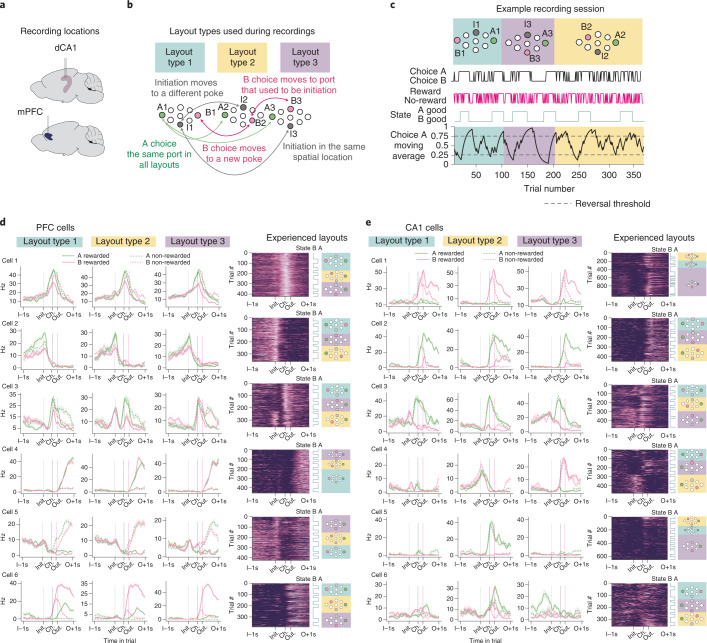
Recording units across multiple problems in a single session. **a**, Silicon probes targeting hippocampal dorsal CA1 and mPFC were implanted in separate groups of mice. **b**, Diagram of problem layout types used during recording sessions. **c**, Example recording session in which a subject completed four reversals in each of three problems. Top panel shows the ports participating in each problem color-coded by layout type. Bottom panel shows the exponential moving average of choices, with the choices, outcomes and reversal blocks shown above. **d**, Example PFC neurons. Cell 1 in PFC fired selectively to both choice ports (but not initiation) in each problem, even though the physical location of the choice ports was different both within and across problems. Cell 2 fired at the initiation port in every problem, even when its physical location changed. Cell 3 fired at B choice ports in all problems but also gained a firing field when initiation port moved to the previous B choice port (showing that PFC does have some port-specific activity). Cell 4 responded to reward at every choice port in every problem. Cell 5 responded to reward omission and had high firing during the ITI. Cell 6 responded to reward at B choice port (that switched location) in each problem. **e**, Example CA1 neurons. Some CA1 cells also had problem general firing properties (cells 1 and 2). Cell 1 fired at B choice that switched physical location between problems. Cell 2 responded to the same port in all problems and modulated its firing rate depending on whether it was rewarded or not. Cell 3 fired at the same port in all layouts. Cell 4 switched its firing preference from initiation to B choice that shared physical locations, analogous to ‘place cells’ firing at a particular physical location. This port selectivity was more pronounced in CA1 than PFC (Extended Data Fig. 4). Cells 5 and 6 ‘remapped’, showing interactions between problem and physical port. Cell 5 fired at a given port in one layout but not when the same port was visited in a different layout. Cell 6 fired at choice time at a given port in one layout and changed its preferred firing time to pre-initiation in a different layout. In all plots, average firing rates are arranged by layout types 1, 2 and 3, but the order in which they were experienced is plotted in the ‘Experienced layouts’ sub-panel. Error bars show firing rates ± s.e.m. across trials.

We recorded single units from dorsal CA1 (345 neurons, *n* = 3 mice, 91–162 neurons per mouse) and mPFC (556 neurons, *n* = 4 mice, 117–175 neurons per mouse) (Supplementary Fig. 1 and Fig. 2) in separate animals using electrophysiology. For recordings, we modified the behavioral task such that changes from one problem to the next occurred within session, with the problem transition triggered once subjects completed four reversals on the current problem, up to a maximum of three problems in one session. Subjects adapted well to this and, in most recording sessions, performed at least four reversals in three problems, allowing us to track the activity of individual units across problems (Fig. 2c). Cross-problem learning reached asymptote before starting recordings—that is, during recording sessions, mice no longer showed improvement across problems (Extended Data Fig. 2), and there were no differences in behavioral performance between CA1 and PFC animals (Extended Data Fig. 2c,f).

During recording sessions (7–16 sessions per mouse, 341–650 trials per session), we used ten different port layouts, but, to simplify the analysis, they were all reflections of three basic layout types (Fig. 2b), each of which occurred once in every session in a randomized order. In the first layout type, the initiation port (I1) was the top or bottom port, and the choice ports were the far left and far right ports. One of these choice ports remained in the same location in all three layouts used in a session and will be referred to as the A choice. This acted as a control for physical location, allowing us to assess how the changing context of the different problems affected the representation of choosing the same physical port. Both the other choice port (B choice) and the initiation port moved physical locations between problems. In the second layout type, both the initiation port (I2) and the B choice port (B2) were in locations not used in layout type 1. In the third layout type, the initiation port was the same as the initiation port in layout type 1 (I3 = I1), and the B choice port was the same as the initiation port from layout type 2 (B3 = I2). Hence, in every recording session, we had examples of (1) the same port playing the same role across problems, (2) different ports playing the same role across problems and (3) the same port playing different roles across problems.

**Fig. 3:**
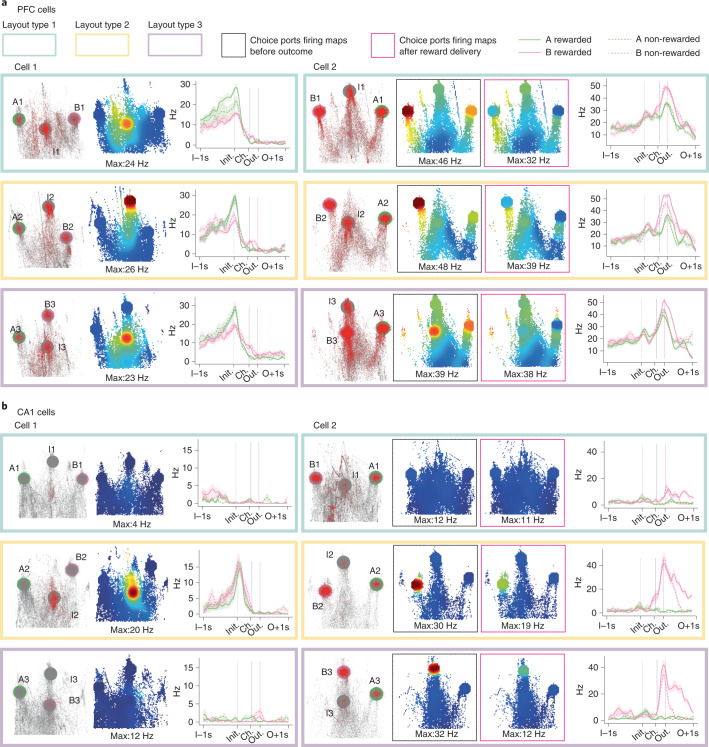
Example neurons in physical space and behavioral task. **a**, Example PFC neurons. For each cell, left panels show trajectories of animals’ nose (gray) and locations where spikes occurred (red) in a 2D space corresponding to the view of a camera positioned above the box looking at the ports, affine transformed to correct for the oblique view of the ports. Middle panels show firing rate heat maps in this same 2D space. Right panels show average firing rates across the trial for each trial type. Layout types are indicated by the color of boxes (blue, yellow and purple). Cell 1 fired at the initiation port in every problem even when its physical location changed. Cell 2 fired at all choice ports in all problems. For choice port selective cells (PFC and CA1 cell 2), we split the firing rate maps by whether the within-choice port spikes (and occupancies) occurred at times before outcome (left) or during reward consumption (right) to further show that these cells are selective to trial events. **b**, Example CA1 neurons. Cell 1 fired at the bottom initiation port in layout type 2 but not when this same port acted as a B choice in layout type 3 or when the port was not a part of the current problem but was visited in layout 1. Cell 2 fired at one of the B ports in one layout type 3 and had no selectivity to the same port in layout type 2 when this port was an initiation port and, instead, fired at a different B choice in layout type 2. Error bars show firing rates ± s.e.m. across trials.

**Extended Data Fig. 3:**
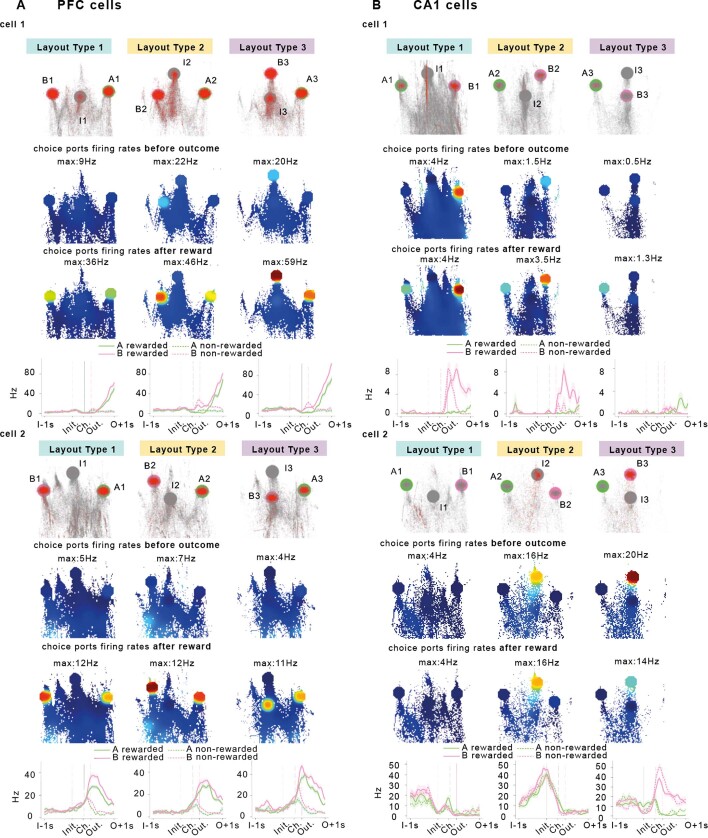
Additional example units in physical space and task. For every cell, the top subpanels show nose trajectories in grey and spikes in red in each problem layout, in a 2D space corresponding to the view of a camera positioned above the box looking at the ports, affine transformed to correct for the oblique view of the ports, (initiation port is indicated in grey, A ports in green and B ports in pink). Middle panels show firing rate heat maps, showing activity within choice ports separately for time before outcome information is delivered, and during reward consumption. Bottom panels show corresponding task event aligned activity. **a) PFC cells**. Cell 1 is a reward cell and fires at all choice ports during the reward consumption period. Cell 2 is a rewarded choice cell and starts to fire for all rewarded choices before the reward is released. **b) CA1 cells**. Cell 1 has a conjunction of space and reward, only firing for B-rewards at ports in the upper right portion of the map. Note that in layout 1 the animal does in fact make some error pokes into a port that is inactive, but will be the B port in layout 2. It fires at this port in layout 2 (where it gets a reward) but not layout 1. Cell 2 is a port selective cell that always fires at the same port, no matter whether it is choice or initiation.

**Extended Data Fig. 4:**
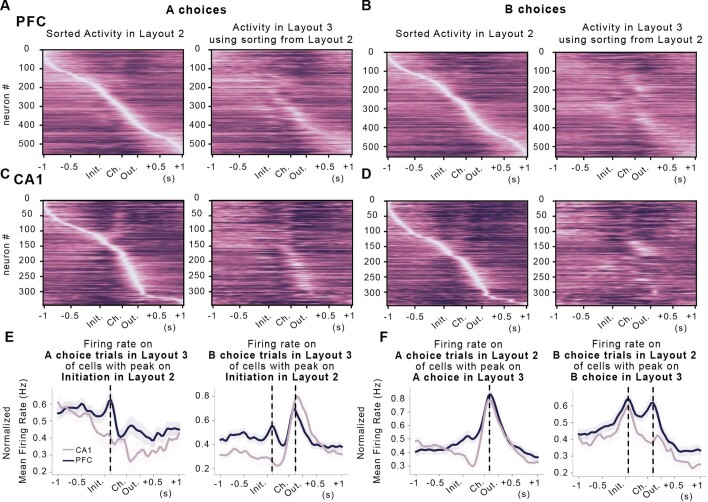
Port selectivity is more pronounced in CA1 than PFC. **a-d)** To evaluate the relative influence of problem-general and port-specific representations in situations where they conflicted, we sorted neurons by their time of peak activity in Layout Type 2 and plotted their activity in Layout Types 2 and 3. The B Choice port in Layout Type 3 was the Initiation port in Layout Type 2. For comparison we also plotted A choices that shared the same physical port in both layouts. **A)** PFC activity on A choice trials in Layout Type 2 (left) and Layout Type 3 (right). **b)** PFC activity on B choice trials in Layout Type 2 (left) and Layout Type 3 (right). **c-d)** Same as **A-B** but for CA1. **E)** We identified cells that had their peak firing rate around initiation in Layout Type 2 and plotted their average activity in Layout Type 3 on A (left) and B (right) choice trials. **f)** We identified cells that had their peak firing rate around A (left) or B (right) choices in Layout Type 3 and plotted their average activity in Layout Type 2 on A (left) and B (right) choice trials. CA1 neurons that fired at initiation in Layout Type 2 fired at B choice in Layout type 3, and vice versa, indicating that they primarily represented physical port. PFC neurons that fired at initiation time in Layout Type 2 generalised to initiation time in layout type 3 but also had a peak at B choice, and vice versa, indicating influence of both task-general and port-specific representations. Error bars on all plots report mean firing rates ± SEM across cells.

As animals transferred knowledge of the trial structure across problems, we reasoned that neurons might exhibit ‘problem-general’ representations of the abstract stages of the trial (initiate, choose and outcome) divorced from the sensorimotor specifics of each problem. On inspection, such cells were common in PFC (Figs. 2d and 3a and Extended Data Fig. 3a). Although some problem-general tuning was observed in CA1, activity for a given trial event (for example, initiation) typically varied more across problems in CA1 than in PFC (Figs. 2e and 3b and Extended Data Figs. 3b and 4). Some CA1 neurons fired at the same physical port across problems even though its role in the task had changed. Other CA1 neurons ‘remapped’ between problems, changing their tuning with respect to both physical location and trial events.

These single-unit examples suggest that problem-general representations may be more prominent in PFC, while both tuning to physical location, and complete remapping between problems may be more common in CA1.

### Representations generalize more strongly in PFC than CA1

To assess whether our single-unit observations hold up at the population level, we sought to characterize how neural activity in each region represented trial events and how these representations generalized across problems.

**Fig. 4:**
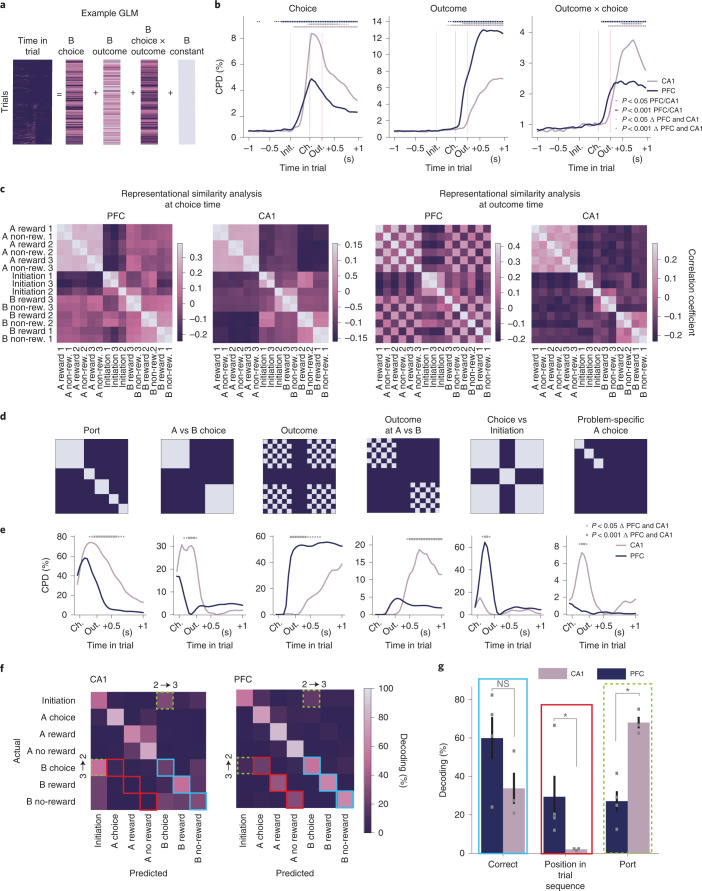
Problem-general and problem-specific representations in PFC and CA1 population activity. **a**, Linear regression predicting activity of each neuron at each timepoint across the trial, as a function of the choice, outcome and outcome × choice interaction. **b**, CPDs from the linear model shown in **a** for choice, outcome and outcome × choice regressors in PFC and CA1. Significance levels for within-region effects were based on a two-sided permutation test where firing rates were rolled with respect to trials. Significance levels for differences between regions were based on a two-sided permutation test across sessions. All significance levels were corrected for multiple comparison over timepoints. **c**, Representation similarity at ‘choice time’ (left) and ‘outcome time’ (right), quantified as the Pearson correlation between the demeaned neural activity vectors for each pair of conditions. **d**, RDMs used to model the patterns of representation similarity observed in the data. Each RDM codes the expected pattern of similarities among categories in **c** under the assumption that the population represents a given variable. The Port RDM models a representation of the physical port poked (for example, far left) irrespective of its meaning in the trial. A vs B choice models a representation of A/B choices irrespective of physical port. The Outcome RDM models representation of reward versus reward omission. The Outcome at A vs B RDM models separate representations of reward versus omission after A and B choices. Choice vs Initiation models representation of the stage in the trial. Problem-specific A choice models separate representation of the A choice in different problems. **e**, CPDs in a regression analysis modeling the pattern of representation similarities using the RDMs shown in **d**. The time course is given by sliding the windows associated with choices from being centred on choice port entry to 0.76 seconds after choice port entry while holding time windows centred on trial initiations fixed. Stars indicate timepoints where regression weight for each RDM was significantly different between the two regions (*P* < 0.05 (small stars) and *P* < 0.001 (big stars)), from one-sided permutation tests across sessions corrected for multiple comparison over timepoints. **f**, Confusion matrices from linear decoding of position in trial, using a decoder that was trained on one problem and tested on another, averaged across animals and across all problem pairs. Colored squares indicate three possible patterns of decoding that indicate different neuronal content. Blue indicates correct cross-task decoding to the same abstract state (for example, B choice decodes to B choice). Red indicates decoding to a different state that could have occurred at the same sequential position in the trial (for example, B choice decodes to A choice). Dashed green corresponds to decoding to the same physical port for those training and test layouts where the Initiation and B choice ports interchanged (for example, B choice decodes to Initiation when the decoder was trained on layout 2 and tested on layout 3). **g**, Bar plots showing the probability of the cross-task decoder outputting the correct abstract state (blue), the other state that can have the same position in the trial sequence (red) and the state that has the same physical port as the training data (dashed green, computed only from confusion matrices where B choice and initiation ports interchange) computed using the corresponding cells highlighted in **f**. Error bars report the mean ± s.e.m. across different mice (CA1: *n* = 3 mice; PFC: *n* = 4). Significance levels were compared against the null distribution obtained by shuffling animal identities between regions (one-sided permutation tests). NS, not significant.

We first assessed the influence of different trial variables in each region using linear regression to predict spiking activity of each neuron, at each timepoint across the trial, as a function of the choice, outcome and outcome × choice interaction on that trial (Fig. 4a). As the task was self-paced, we aligned activity across trials by warping the time period between initiation and choice to match the median interval (for more details, see ‘Time warping methods’ and Supplementary Fig. 2). We then quantified how strongly each variable affected population activity as the population coefficient of partial determination (CPD) (that is, the fraction of variance uniquely explained by each regressor) at every timepoint across the trial (Fig. 4b). This analysis was run separately for each problem in the session, and the results were averaged across problems and sessions. Both regions represented current choice, outcome and choice × outcome interaction, but there was regional specificity in how strongly each variable was represented. Choice (A vs B) representation was more pronounced in CA1 than PFC (peak variance explained—CA1: 8.4%, PFC: 4.8%, *P* < 0.001), whereas outcome (reward vs no reward) coding was stronger in PFC (peak variance explained—CA1: 7.1%, PFC: 12.9%, *P* < 0.001). Furthermore, choice × outcome interaction explained more variance in CA1 than PFC (peak variance explained—CA1: 3.7%, PFC: 2.4%, *P* < 0.001).

Although highlighting some differences in population coding between regions, this approach cannot assess the relative contribution of abstract representations that generalize across problems versus features specific to each problem, such as the physical port location. This requires comparing activity both across timepoints in the trial and across problems, which we did using representational similarity analysis (RSA)^44^. We extracted firing rates around initiation and choice port entries (±20 ms around each port entry type) and categorized these windows by which problem they came from, whether they were initiation or choice, and, for choice port entries, whether the choice was A or B and whether it was rewarded, yielding a total of 15 categories (Fig. 4c). For each session, we computed the average activity vector for each category and then quantified the similarity between categories as the correlation between the corresponding activity vectors. We show RSA matrices for this ‘choice time’ analysis (Fig. 4c, left panels) and an ‘outcome time’ analysis (Fig. 4c, right panels) where the windows for choice events were moved 250 ms after port entry, holding the time window around trial initiations constant.

To quantify the factors influencing representation similarity, we created representational similarity design matrices (RDMs) that each encapsulated the predicted pattern of similarities under the assumption that activity was influenced by a single feature of the behavior (Fig. 4d). For example, if the population activity represented only which physical port the animal was at, its correlation matrix would look like Fig. 4d, Port. We included RDMs for a set of problem-general features: the trial stage (‘Initiation vs Choice’), choice (A vs B) and trial outcome (both on its own as ‘Outcome’ and in conjunction with choice ‘Outcome at A vs B’). To assess whether the changing context provided by different problems modified the representation of choosing the same physical port at the same trial stage, we included a ‘Problem-specific A choice’ RDM that represents similarity between A choices (which are always in the same location) within each problem.

To assess the influence of these features on neural activity, we modeled the experimentally observed patterns of representational similarity (Fig. 4c) as a linear combination of the RDMs (Fig. 4d), quantifying the influence of each by its corresponding weight in the linear fit. As the RSA matrices changed between choice time and outcome time (Fig. 4c), we characterized this time evaluation using a series of such linear fits, moving the time window around choice port entries in steps from before port entry until after the reward delivery while holding the time window around initiation port constant, generating the time series for the influence of each RDM on activity shown in Fig. 4e.

Consistent with our single-unit observations, both PFC and CA1 represented both problem-specific and problem-general features to some extent. However, there was a marked regional specificity in how strongly different features were encoded (Fig. 4e). PFC had stronger, abstract, sensorimotor-invariant representation of trial stage (Initiation vs Choice) and trial outcome (*P* < 0.001). In contrast, CA1 had stronger representation of the physical port that the subjects were poking and whether it was an A vs B choice (*P* < 0.001). Additionally, CA1, but not PFC, showed a problem-specific representation of A choices (*P* < 0.001). This is striking because, during A choices, both the physical port and its meaning are identical across problems, indicating that the changing problem context alone induced some ‘remapping’ in CA1 but not PFC. Finally, there was a regional difference in the representation of trial outcome. PFC outcome representations were more general (the same neurons responded to reward or reward omission across ports and problems, *P* < 0.001). CA1 also maintained an outcome representation, but this was more likely to be conjunctive than in PFC—different neurons would respond to reward on A and B choices (*P* < 0.001).

**Extended Data Fig. 5:**
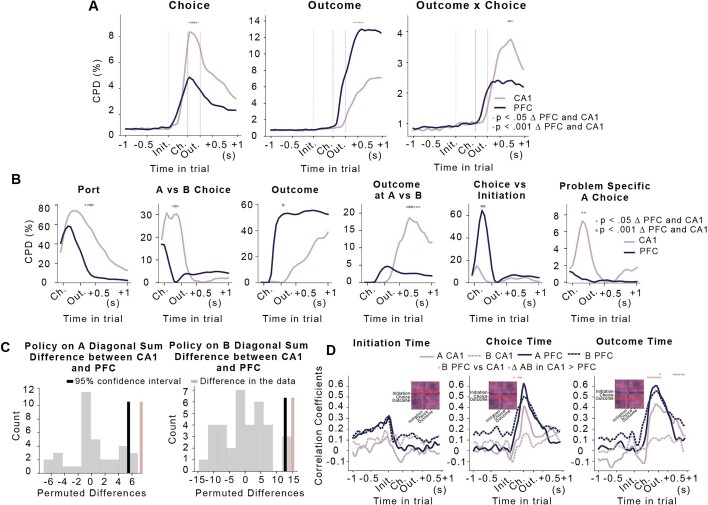
Animal based permutation tests. Significance of key differences between CA1 and PFC assessed by permuting subjects between regions, rather than sessions as done in main figures. **a)** Coefficients of partial determination from the linear model shown in Fig. 4a for choice, outcome, and outcome x choice regressors in PFC and CA1. **b)** Coefficients of partial determination in a regression analysis modelling the pattern of representation similarities using the RDMs shown in Fig. 4d. **c)** Sums along the diagonal of the correlation matrices shown in Fig. 6c separately for A and B choices. **d)** Slices through the correlation matrices at initiation (left), choice (centre) and outcome (right) times for A (solid) and B (dash line) choices. For animal shuffles in singular value decomposition analyses see Extended Data Fig. 9.

**Extended Data Fig. 6:**
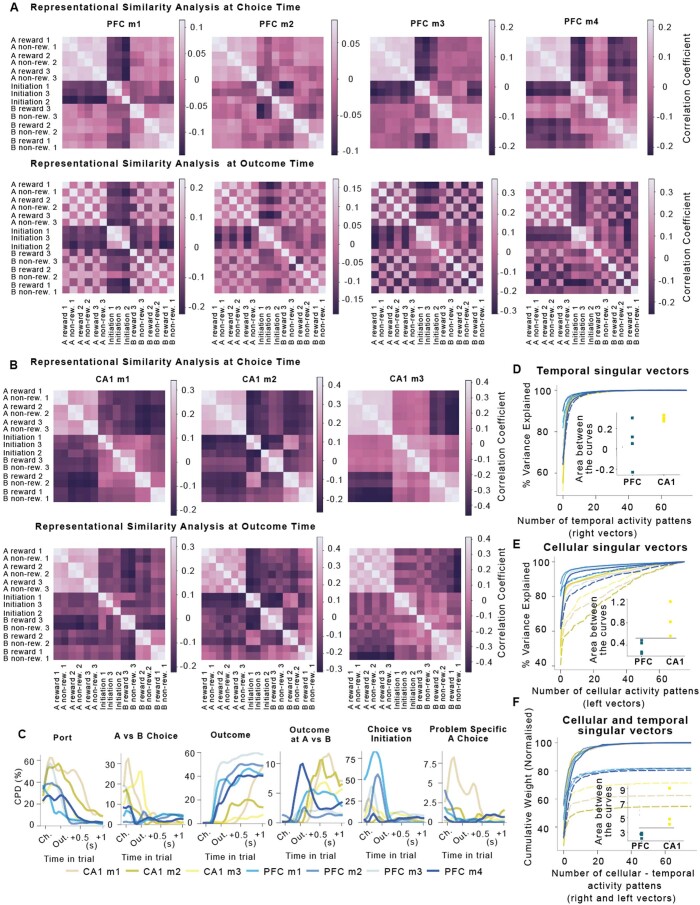
RSA and the low dimensional structure of activity analyses for individual animals. **a)** Representation similarity at 'choice time' (top) and 'outcome time' (bottom) for each PFC mouse, quantified as the Pearson correlation between the demeaned neural activity vectors for each pair of conditions. **b)** Representation similarity at 'choice time' (top) and 'outcome time' (bottom) for each CA1 mouse. **c)** Coefficients of partial determination (CPDs) in regression analyses modelling the patterns of representation similarities in individual mice using the RDMs shown in Fig. 4d. **D)** Variance explained when using temporal activity patterns *V*_*1*_^*T*^ from one problem to predict either held out activity from the same problem (solid lines) or activity from a different problem (dash lines) in individual PFC and CA1 mice. **e)** Variance explained when using cellular activity patterns *U*_*1*_ from one problem to predict either held out activity from the same problem (solid lines) or activity from a different problem (dash lines) in individual PFC and CA1 mice. **f)** Cumulative weights along the diagonal Σ using pairs of temporal *V*_*1*_^*T*^ and cellular *U*_*1*_ activity patterns from one problem to predict either held out activity from the same problem (solid lines) or activity from a different problem (dash lines) in individual PFC and CA1 mice. Subpanels in D, E and F show differences in area under the curve (within - between problems) for each CA1 and PFC animal.

**Extended Data Fig. 7:**
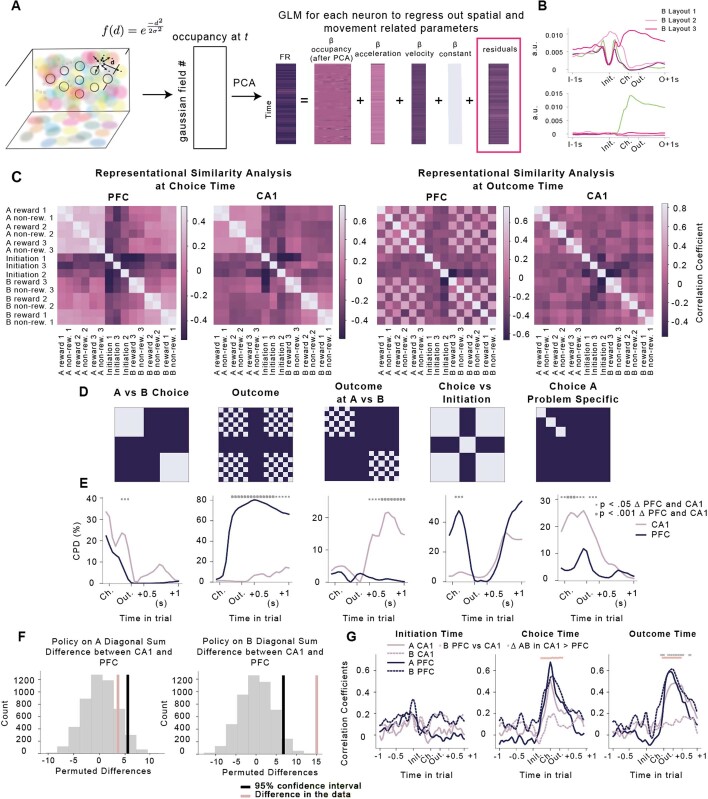
Fine-grained movement related activity controls. **a)** We do not expect 2D nose coordinates to be linearly related to firing rates so to account for place cell like coding of nose position in the firing rates of neurons we defined a set of gaussian “radial basis functions” with the centres randomly selected from an animal’s 2D coordinates in each session (different coloured circles). Next, for each time point we calculated the activity of each basis function (gaussian in distance from centre of this field) resulting in a time x (# of basis functions) matrix (left). To account for cross-correlations in this matrix we next did a principal component analysis to extract the first ten orthogonal occupancy components across time (middle). Next, we fit a linear regression model predicting firing rates of neurons with occupancy as well as nose velocity, and acceleration predictors, resulting in residual firing rates that do not contain variability related to these movement and position parameters (right). **b) Top two principal components from the PCA analysis of occupancies in A from an example session**. The first component differentiates initiation in Layout 1 and port B in Layout 2 (same physical location) from other ports. The second component differentiates port A (same physical location) from all other ports. **c)** Representation similarity at 'choice time' (left) and 'outcome time' (right), quantified as the Pearson correlation between the **residual** neural activity (after accounting for movement related parameters) vectors for each pair of task conditions as in Fig. 4c. **d)** Representational Similarity Design Matrices (RDMs) used to model the patterns of representation similarity observed in the data. Port RDM was not included in this analysis as the PCs we regress out to account for movement related activity correlate strongly with the port position in the task (as in **b**). Using an RDM that is so highly correlated with a parameter that has already been regressed out can lead to false correlations (a la Berkson’s paradox). **e)** Coefficients of partial determination in a regression analysis modelling the pattern of representation similarities in residual firing rates using the RDMs in D. **f-g)** Policy analyses conducted on residual firing rates after accounting for movement related activity. The generalisation of policy on A trials is no longer stronger in PFC than CA1. Stars denote significance levels from two-sided permutation tests across sessions corrected for multiple comparison over time points. Controls of precise physical movements in singular value decomposition analyses are presented in the main text (Fig. 5d–j).

**Extended Data Fig. 8:**
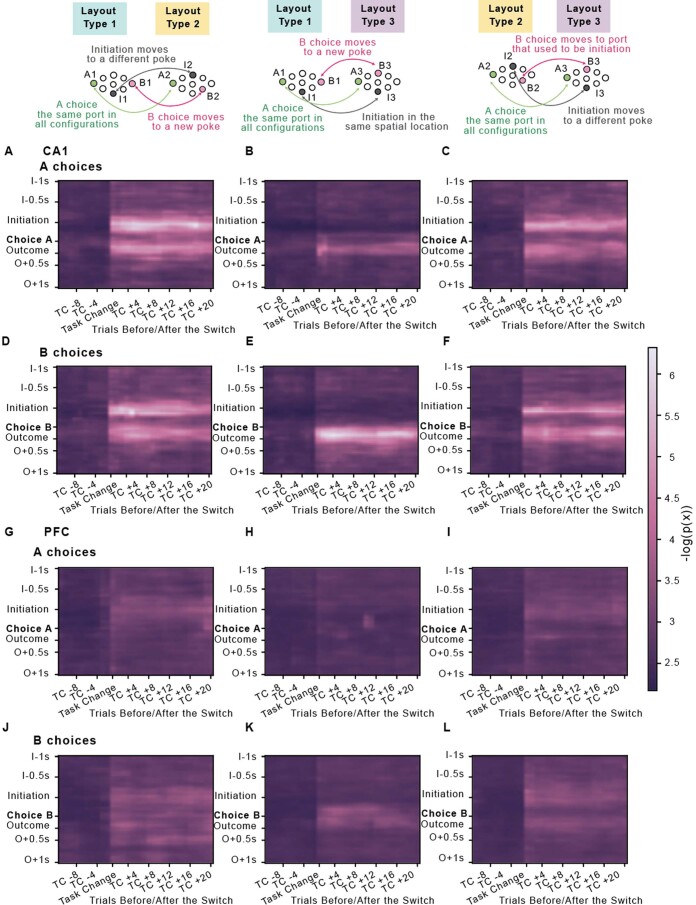
Rapid problem-induced ‘remapping’ in CA1 but not PFC. The change in activity across transitions between problems was quantified using a ‘surprise’ measure indicating how unexpected the population activity was on each trial and time-point given the average activity at that time-point across 10 ‘baseline’ trials prior to those shown in the figure (see *Surprise Measure Methods*). Three types of transition between Layout Types were analysed (left, middle and right columns), the diagrams at the top of the figure show how initiation and choice ports changed position for each. In the first type of transition (left) both initiation and B choice were in different locations in the two problems. In the second type (middle) initiation was in the same physical location but B choices were in different ports. In the third type (right) initiation was in different physical locations but initiation port in layout 2 was in the same location as choice B in layout 3. The A choice port was always in the same physical location in all problem layouts. **a-j)** Heatmaps showing how surprising the activity at each time point of each trial was around transitions between problems. Activity in CA1 on A and B choice trials is shown in **a-c** and **d-f** respectively. Activity in PFC on A and B choice trials is shown in **g-i** and **j-l** respectively. In CA1, when an initiation or choice port moved to a different physical location, the neuronal representation at the corresponding stage of the trial changed immediately, as indicated by an abrupt increase in surprise at the layout transition **a-f**.

These representational differences between regions survived the animal random effects test (see the ‘Statistical significance’ section, Extended Data Fig. 5 and individual animal plots in Extended Data Fig. 6a–c). To ensure that they were not driven by fine-grained selectivity to physical movements, we re-ran the analysis on residual firing rates after regressing out the influence of two-dimensional (2D) nose position, velocity and acceleration (for more details, see the ‘Additional controls for physical movement’ section). All inter-region differences except the stronger representation of A vs B choice in CA1 survive this control (Extended Data Fig. 7c–e), consistent with the single-cell examples described above (Fig. 3a,b and Extended Data Fig. 3). We also assessed whether problem specificity in CA1 might be driven by slow drift over time but found that representations changed abruptly at transitions between problems (Extended Data Fig. 8).

We used a cross-problem decoding analysis to further characterize differences in representation between regions. We trained a linear model to decode position in the trial (Initiation and A/B choice/reward/no-reward) using data from one problem and tested the decoding performance on a different problem (Fig. 4f,g). Because the B and initiation ports moved and sometimes interchanged between problems, the pattern of decoding errors is informative about whether activity primarily represented physical port or abstract trial stage (Initiation vs Choice). Where PFC made errors, they were predominantly to the other state that could occur at the same sequential position in the trial (A rather than B choice or outcome). By contrast, CA1 predominantly decoded to the same physical port as the training data. Together, these population results confirm that PFC had a predominantly generalizing representation, and this representation embeds the sequential properties of the trial while CA1 encoded problem specifics (such as port identity) more strongly.

### Generalization of low-dimensional population activity

To further explore how the structure of population activity generalized between problems, we assessed how accurately low-dimensional activity patterns in one problem could explain activity in another. Using singular value decomposition (SVD), we decomposed activity in each problem into a set of cellular and temporal modes. Cellular modes correspond to sets of neurons whose activity covaries over time and, hence, can be thought of as cell assemblies. Each cellular mode is specified by a vector with a weight for each cell, indicating how strongly the cell participates in the mode. Cellular and temporal modes come in pairs, such that each cellular mode has a corresponding temporal mode, which is a vector of weights across timepoints indicating how the activity of the cellular mode varies over time.

**Fig. 5:**
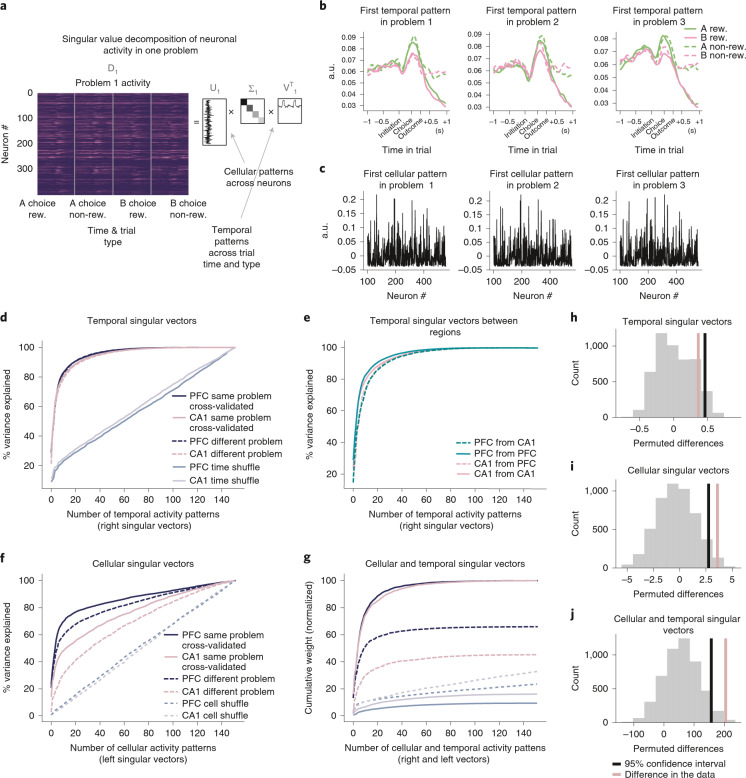
Generalization of low-dimensional representations of trial events. **a**, Diagram of SVD analysis. A data matrix comprising the average activity of each neuron across timepoints and trial types was decomposed into the product of three matrices, where diagonal matrix Σ linked a set of temporal patterns across trial type and time (rows of *V*^*T*^) to a set of cellular patterns across cells (columns of *U*). **b**, First temporal mode in *V*^*T*^ from SVD decomposition of data matrix from PFC plotted in each problem separately for clarity and separated by A (green) and B (pink) rewarded (solid) and non-rewarded (dashed) choices. **c**, First cellular mode from SVD decomposition of data matrix from PFC in each problem showing that similar patterns of cells participate in all problems. **d**, Variance explained when using temporal activity patterns *V*_*1*_^*T*^ from one problem to predict either held-out activity from the same problem (solid lines) or activity from a different problem (dashed lines). Light purple and lilac lines indicate variance explained when shuffling timepoints in the firing rates matrices. **e**, Variance explained when using temporal activity patterns *V*_*1*_^*T*^ to predict either activity from the same problem and brain region (solid lines) or a different brain region (and, therefore, different animal) and the same problem (dashed lines) *D*_*2*_. **f**, Variance explained when using cellular activity patterns *U*_*1*_ from one problem to predict either held-out activity from the same problem (solid lines) or activity from a different problem (dashed lines). Dashed light purple and lilac lines indicate variance explained when shuffling cells in the firing rates matrices. **g**, Cumulative weights along the diagonal Σ using pairs of temporal *V*_*1*_^*T*^ and cellular *U*_*1*_ activity patterns from one problem to predict either held-out activity from the same problem (solid lines) or activity from a different problem (dashed lines). Weights were normalized by peak cross-validated cumulative weight computed on the activity from the same problem. **h**, To assess whether the temporal singular vectors generalized significantly better between problems in PFC than CA1, we evaluated the area between the dashed and solid lines in **d** for CA1 and for PFC separately, giving a measure for each region of how well the singular vectors generalized. We computed the difference in this measure between CA1 and PFC (pink line in **h**) and compared this difference to the null distribution obtained by permuting sessions between brain regions (gray histogram; black line shows the 95th percentile of distribution). Temporal singular vectors generalized equally well between problems in the two regions. **i**, Cellular singular vectors generalized significantly better between problems in PFC than CA1. Computed as in **h** but using the solid and dashed lines from **f**. **g**, Pairs of cellular and temporal singular vectors generalized significantly better between problems in PFC than CA1. Computed as in **h** but using the solid and dashed lines from **g**. a.u., arbitrary units.

To evaluate the cellular and temporal modes for a given problem, we first regressed out general movement-related features onto the firing rates (for more details, see Extended Data Fig. 7 and the ‘Additional controls for physical movement’ section). After removing the effect of velocity, acceleration and 2D nose position, we computed the average residual firing rate at each timepoint across the trial for four types of trials: rewarded A choices, non-rewarded A, rewarded B and non-rewarded B (non-rewarded trials included both correct trials and incorrect trials). For each cell, we concatenated these four time series to create a single time series containing the average activity of the cell across each timepoint of the four trial types. The temporal modes span this same set of timepoints and, hence, capture variation across both time-in-trial and trial-type. We then stacked these single-cell activity time series for all neurons to create an activity matrix *D* where each row contained the activity of one neuron (Fig. 5a). Using SVD, we decomposed this activity matrix into cellular and temporal modes *U* and *V*, linked by a diagonal weight matrix Σ

$$D = U{\Sigma} V^T$$

The cellular modes are the columns of *U*, and the temporal modes are the rows of *V*^*T*^. Both modes are unit vectors, so the contribution of each pair to the total data variance is determined by the corresponding element of the diagonal matrix Σ. The modes are sorted in order of explained variance, such that the first cellular and temporal mode pair explains the most variance. The first cellular and temporal mode of PFC activity in three different problems is shown in Fig. 5b,c. It is high throughout the inter-trial interval (ITI) and trial, with a peak at choice time but strongly suppressed after reward (similar to cell 5 in Fig. 2d).

We reasoned that (1) if the same events were represented across problems (for example, initiation, A/B choice and outcome), then the temporal modes would be exchangeable between problems, no matter whether these representations were found in the same cells; (2) if the same cell assemblies were used across problems, then the cellular modes would be exchangeable across problems, no matter whether the cell assemblies played the same role in each problem; and (3) if the same cell assemblies performed the same roles in each problem, then pairs of cellular and temporal modes would be exchangeable across problems.

To see whether the same representations existed in each problem, we first asked how well the temporal modes from one problem could be used to explain activity from other problems. Because the set of temporal modes *V* is an orthonormal basis, any data of the same rank or less can be perfectly explained when using all the temporal modes. However, population activity in each problem is low dimensional, so a small number of modes explain a great majority of the variance. Modes that explain a lot of variance in one problem will explain a lot of variance in the other problem only if the structure captured by the mode is prominent in both problems. The question is, therefore, how quickly variance is explained in problem 2ʼs data, when using the modes from problem 1 ordered according to their variance explained in problem 1. To assess this, we projected the data matrix *D*_*2*_ from problem 2 onto the temporal modes *V*_*1*_ from problem 1, giving a matrix *M*_*V*_ whose elements indicate how strongly each temporal mode contributes to the problem 2 activity of each neuron:

$$M_V = D_2V_1$$

The variance explained by each temporal mode is given by squaring the elements of *M*_*V*_ and summing over neurons. We express this as a percentage of the total variance in *D*_*2*_ and plot the cumulative variance explained as a function of the number of *D*_*2*_ʼs temporal modes, when ordering modes according to variance explained in *D*_*1*_ (Fig. 5d). To control for drift in neuronal representations across time, we computed the data matrices separately for the first and second halves of each problem. We compared the amount of variance explained using modes from the first half of one problem to model activity in the second half of the same problem, with the variance explained using modes from the second half of one problem to model activity from the first half of the next problem.

In both PFC and CA1, the cumulative variance explained as a function of the number of temporal modes used did not depend on whether the two datasets were from the same problem (solid) or different problems (dashed) (Fig. 5d,h; *P* > 0.05). This indicates that the temporal patterns of activity and, therefore, the trial events represented did not differ across problems in either brain area. However, as this analysis used only the temporal modes, it says nothing about whether the same or different neurons represented a given event across problems. In fact, we can even explain activity in one brain region using temporal modes from another region and mouse (Fig. 5e).

The pattern was very different when we used cellular modes (that is, assemblies of co-activating neurons) from one problem to explain activity in another. We quantified variance explained in problem 2 using cellular modes from problem 1, by projecting the problem 2 data matrix *D*_*2*_ onto problem 1 cellular modes *U*_1_, giving a matrix *M*_*u*_ whose elements indicate how strongly each cellular mode contributes to problem 2 the activity at each timepoint:

$$M_U = U_1^TD_2$$

The total variance explained by each temporal mode is given by squaring the elements of *M*_*U*_ and summing over timepoints. In both PFC and CA1, cellular modes in *U* that explained a lot of variance in one problem explained more variance in the other half of the same problem than they did in an adjacent problem (Fig. 5f; differences between solid and dashed lines). However, the within-problem versus cross-problem difference was larger in CA1 than PFC (Fig. 5i; *P* < 0.05). This indicates that PFC neurons whose activity covaried in one problem were more likely to also covary in another problem, when compared to CA1 neurons. As this analysis considered only the cellular modes, it does not indicate whether a given cell assembly carried the same information across problems.

To assess how well the cellular and temporal activity patterns from problem 1 explained activity in problem 2, we projected dataset *D*_2_ onto the cellular and temporal mode pairs of problem 1 ($U_1^T$, *V*_*1*_).

$$\Sigma _2 = U_1^TD_2V_1$$

**Extended Data Fig. 9:**
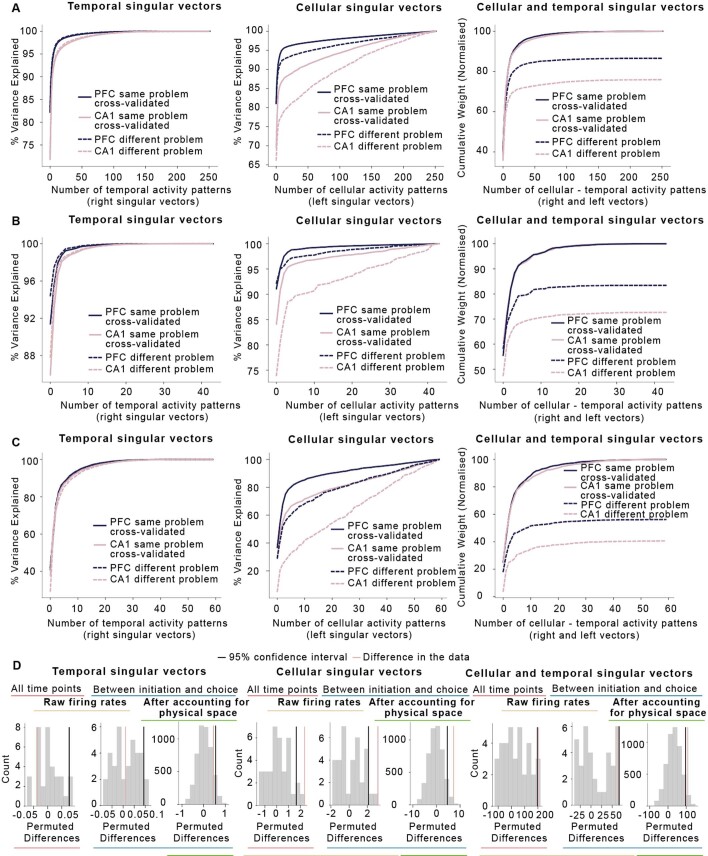
Additional analyses of low dimensional structure of activity in PFC and CA1. **a – c)** Generalization of low dimensional structure of activity across problems when considering; **a**) all time-points in the trial (raw firing rates), **b**) only time-points between initiation and choice (raw firing rates), **c**) only time-points between initiation and choice using residual firing rates after accounting for physical movement. Left: Variance explained when using temporal activity patterns *V*_*1*_^*T*^ from one problem to predict either held out activity from the same problem (solid lines) or activity from a different problem (dash lines). Middle: Variance explained when using cellular activity patterns *U*_*1*_ from one problem to predict either held out activity from the same problem (solid lines) or activity from a different problem (dash lines). Right: Cumulative weights along the diagonal Σ using pairs of temporal *V*_*1*_^*T*^ and cellular *U*_*1*_ activity patterns from one problem to predict either held out activity from the same problem (solid lines) or activity from a different problem (dash lines). **d)** Permutation tests for significance of differences between CA1 and PFC in generalization of temporal (left), cellular (middle) and cellular and temporal (right) singular vector, based on the null distribution obtained by shuffling animals across groups (raw firing rates) or sessions (residual firing rates after accounting for physical space). We could not permute animals in the analyses of residual firing rates because we were not set up for recording video data for our first implanted animal.

If the same cell assemblies perform the same roles in two different problems, the temporal and cellular modes will align, and Σ_2_ will have high weights on the diagonal. We, therefore, plotted the cumulative squared weights of the diagonal elements of Σ within and between problems (Fig. 5g). In both PFC and CA1, cellular and temporal modes aligned better in different datasets from the same problem (solid lines) than for different problems (dashed lines). However, this difference was substantially larger for CA1 than PFC (Fig. 5j; *P* < 0.05). All results also held true when using a time window between only initiation and choice (Extended Data Fig. 9).

These data show that, although the temporal structure of activity in both regions generalizes perfectly across problems, brain regions and subjects— a consequence of the same set of trial events being represented in each—the cell assemblies used to represent them generalized more strongly in PFC than CA1.

### Generalization of policy representations

So far, we have focused on how neuronal representations of individual trial events generalize across problems. But, to maximize reward, the subject must also track which option is best by integrating the history of choices and outcomes across trials. To be useful for generalization, this policy representation should also be divorced from the current sensorimotor experience of any specific problem.

**Fig. 6:**
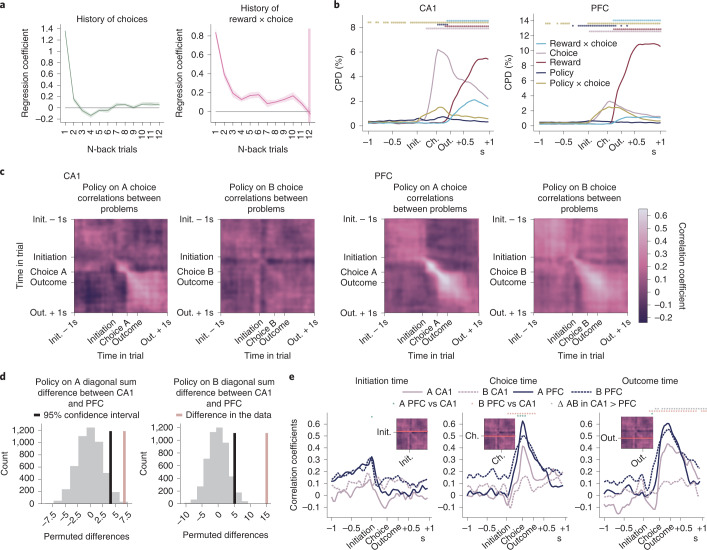
Policy generalization in PFC and CA1. **a**, Weights from logistic regression predicting choices in recording sessions using choices, rewards and choice × reward interactions over the previous 12 trials as predictors. The effect of choice × outcome interaction history was significantly above zero on up to 11 trials back (one-sided *t*-test, *P* < 0.05) except for the 7th trial (*t*_6_ = 1.99, *P* = 0.094). Error bars report the mean ± s.e.m. across mice. **b**, CPDs from regression models predicting neural activity using current trial events, subjects’ policy (estimated using the behavioral regression in **a**) and policy interacted with current choice. Stars denote the timepoints at which each regressor explained significantly more variance than expected by chance (permutation test based on rolling firing rates with respect to trials, *P* < 0.001, corrected for multiple comparisons; for more details on permutation tests, see the ‘Statistical significance’ section). **c**, Correlations across problems between policy weights in regressions predicting neural activity. Regressions were run separately for A (left panels) and B (right panels) choices in each problem and at each timepoint across the trial. Correlations of policy representations between all problem pairs were evaluated for each pair of timepoints; values on the diagonal show how correlated policy representation was at the same timepoint in both problems. Positive correlation indicates that the same neurons coded policy with the same sign in both problems. **d**, To quantify whether policy generalized more strongly between problems in PFC than CA1, we computed the between-region difference in the sum along the diagonal of the correlation matrices in **c**, separately for A and B choices, and compared it against the null distribution obtained by permuting sessions between brain regions. Policy representation on both A and B choices generalized more strongly in PFC than CA1. **e**, Slices through the correlation matrices at initiation (left), choice (center) and outcome (right) times for A (solid) and B (dashed line) choices. Significant differences between conditions are indicated by stars as shown in the legend.

To estimate subjects’ beliefs about which option was best, we used a logistic regression predicting the current choice as a function of the choice and outcome history (Fig. 6a). This gave a trial-by-trial estimate of the probability the animal would choose A versus B—that is, the animal’s policy. We used this policy as a predictor in a linear regression predicting neural activity, run separately for each problem with results averaged across problems and sessions (Fig. 6b). Policy explained variance that was not captured by within-trial regressors such as choice, reward and choice × reward interaction. Specifically, the subjects’ policy interacted with the current choice-explained variance (*P* < 0.001) starting around the time of trial initiation, when it would be particularly useful for guiding the decision.

We next asked whether this policy representation generalized across problems. Policy may generalize differentially for A and B choices because only the B port varied between problems. We, therefore, analyzed A and B choice trials separately. We ran a set of linear regressions, each predicting neural activity in one problem at a single timepoint in the trial, using policy and trial outcome as regressors. The policy beta weights from each regression correspond to the pattern of neural activity that represented policy in one problem at one timepoint. We can, therefore, quantify the extent to which policy representations generalized between problems as the correlation coefficient between the policy beta weights. We computed the average across-problem correlation of these weights between every pair of timepoints (Fig. 6c). The diagonal elements of these matrices show the average correlation across problems at the same timepoint in each problem. These correlations were larger in PFC than CA1 on both A and B choices (*P* < 0.05, permutation test; Fig. 6d), showing that, on average, policy representations generalized across problems better in PFC than CA1.

One possible explanation is that PFC simply represented action values in a problem-general way. A more interesting possibility is that current policy shapes the representation of each trial stage differently, but, in CA1, these representations are more tied to the sensorimotor specifics of the current problem. To test this, we examined time slices through the correlation matrices at initiation, choice and outcome times (Fig. 6e). In PFC, all three correlation profiles on both A and B trials peaked at the correct timepoint (the equivalent to the diagonal elements of the matrix)—that is, the policy representations generalized across problems but were specific to each part of the trial (initiate, choose and outcome). A similar pattern was present in CA1 but only on A choices (which are the same physical port across problems). No CA1 correlation was significantly above zero on B choices. Indeed, whereas PFC policy correlations were greater than CA1 correlations for all representations (all *P* < 0.05) on both A and B choices, CA1 correlations showed a greater difference between A and B trials at outcome time (Fig. 6e; all *P* < 0.05).

Overall, therefore, both PFC and CA1 maintained representations of the subject’s current policy that were not simple value representations, as they differed depending on the trial stage. These representations were abstracted across problems in PFC but tied to the sensorimotor specifics in CA1. A portion, but not all, of this problem specificity in CA1 was accounted for by the port identity.

## Discussion

Humans and other animals effortlessly generalize prior experience to novel situations that are only partially related. To do this, we must reduce experiences to abstractions—features that are common between different situations. Critically, we must also bind these abstractions to the specifics of the current situation. Our study makes three contributions to understanding how, and when, this process happens.

First, we show that this focus on abstraction, common in studies of spatial reasoning and memory^2,3^, is also important in standard reinforcement learning paradigms, such as reversal learning. Whereas the dominant focus in these paradigms has been on variables such as value and prediction error^45–47^ (important for learning actions de novo), we show that the neural representation in mPFC reflects the temporal structure of the problem itself, which may allow actions to be generalized from similar previous experiences. One intriguing possibility is that such representations are formed during the shaping process that precedes most operant experiments.

Second, we show that mPFC and CA1 contain different representations that suggest different functional roles. Population responses in mPFC were dominated by problem-invariant representations that might form the abstraction. By contrast, the CA1 responses contained major sources of variance that were either invariant to the sensorimotor particularities (port selective) or, intriguingly, the interaction of these with the problem structure (demonstrating ‘remapping’ between problems or reflecting the interaction of task policy and individual port). Representations such as these are required to bind task-general abstractions to the current sensory problem.

Third, we show that task abstractions in mPFC simultaneously represent behavior over markedly different temporal scales. Part of the mPFC representation pertained to the immediate next action in the sequence (for example, go to the initiation port), but part of the representation pertained to the integrated history of rewards and actions over many trials that allowed the animal to make profitable choices. Notably, both parts of the representation were largely maintained in an abstract form that generalized over problems with different sensory particularities.

These findings are related to previous findings across several different literatures.

In reinforcement learning, recent data have highlighted the low-dimensional structure of abstract task representations in rodent orbitofrontal cortex^9^. This aligns with our finding that low-dimensional temporal modes are consistent across different sensorimotor instances of the reversal learning problem in both mPFC and CA1. We also confirm that they are consistent between animals and further demonstrate that they are broadly consistent between different brain areas (mPFC and CA1), suggesting that this low-dimensional temporal structure does not reflect the unique representational properties of a particular brain area. Notably, however, because we record across the same neurons in different problems, we are able to ask not only whether the temporal dimensions are preserved across problems but also whether these temporal modes align to the same neurons in each problem—that is, whether the same neurons represent the same trial events across problems. They do so significantly more in PFC than CA1. It is this that enables us to propose different functional roles for the two different brain regions.

Recent reinforcement learning work has also found a form of abstraction in primate PFC and hippocampus^48^. Because abstraction was assessed across conditions that used the same physical operandum and, hence, shared sensorimotor correlates, it is not possible in these data to discern whether hippocampal representation would generalize to different sensorimotor instantiations of the same problem. By contrast, the focus of our study is on how these brain regions enable generalization of knowledge across problems that share the same abstract structure but different sensorimotor experiences. In future work, it would be valuable to examine the converse situation, where problems with different abstract structure recruit the same sensorimotor sequences. Such designs would be particularly powerful in contexts where theory makes quantitative predictions for how task structure shapes representations^49,50^.

The essence of reinforcement learning is the integration of rewards over temporally extended experiences to generate expected values or policies^51^. Our demonstration that these policy representations are abstracted aligns directly with ideas from computer science, such as meta-reinforcement learning^6,52,53^, which have recently been proposed as models to understand prefrontal activity. Indeed, our behavioral data directly demonstrate meta-learning, as reversals become faster with increasing experience.

Notably, we also found that policy coding was not unique to PFC, as hippocampus also contained policy representations, corroborating existing findings for the existence of signals relevant for decision-making in hippocampal formation^54–56^. We expand on these observations to provide further evidence that hippocampal activity might represent sensorimotor specifics of events in the context of broader memory schemas and task structures.

Although relatively new to the neuroscience of reinforcement learning, the overarching ideas in our study are central in the study of memory and space. Here, it is commonly assumed that hippocampal representations reflect the sensory details of each episodic experience^19,20,57^, and cortical representations abstract these details to allow generalization^58–60^. Indeed, in spatial studies in rodents, new abstractions (schemas) rely causally on mPFC^3^. Equally, spatial reasoning in rodents is dependent on grid cells^30^, which abstract of the fundamental 2D properties of physical space. Recent data and modeling have shown that hippocampal spatial representations are bound to this abstraction^40,61^. We think that our study demonstrates that many of these ideas carry directly over to structural abstractions in reinforcement learning problems and, therefore, further align these historically distinct fields.

We do not perceive the world as it really is. Starting with the visual 2D inputs on the retina that we use along with prior experience to infer the 3D world around us^62^, our brains likely develop structural placeholders for many of our experiences. In fact, we remember things more easily if we know the general schema or a script for a particular event^63^, and we often ignore information that does not align with our understanding of the world^64^. More broadly, here we demonstrate that mice also acquire sophisticated models of tasks that they frequently experience in their environment and can apply this knowledge to solve new problems faster. We further show that PFC contains generalized representations of variables needed to solve new related problems while hippocampus combines sensorimotor and abstract information to represent an interaction between the two, which might be crucial for both interpreting our ongoing experiences as well as encoding and recall of episodic memories.

## Methods

### Behavioral apparatus

Experiments were performed in custom-made operant boxes, controlled using pyControl^65^. The boxes used in the training phase of the experiment had six nose-poke ports on the back wall, each with infrared beam, stimulus LED and solenoid valve for dispensing liquid rewards and a speaker for auditory stimuli. For recording experiments, mice were transferred to operant boxes with nine nose-poke ports located in electrically shielded sound-attenuating chambers. The operant box design is detailed at https://github.com/pyControl/hardware/tree/master/Behaviour_box_small.

### Subjects

Nine male C57BL/6J mice were used in the study, aged 6 weeks at the start of the experiment. Animals were group-housed before surgery and individually housed after surgery on a 12-hour light/dark cycle. All nine animals were implanted with silicon probes, but we obtained data from only seven, due to one probe being damaged during surgery and having to cull one animal before recordings. No statistical methods were used to predetermine sample sizes, but our sample sizes are similar to those reported in previous publications^9,37,38^. Animals were pseudo-randomly assigned to the CA1 and PFC groups. Data collection and analysis were not performed blinded to the conditions of the experiments. Experiments were carried out in accordance with Oxford University animal use guidelines and performed under UK Home Office Project Licence P6F11BC25.

### Behavioral training

Mice were placed on water restriction 48 hours before starting behavioral training, with 1 hour of water access provided 24 hours before the first session. Mice were trained 6 days per week, and, on the day off, they received 1 hour ad libitum water access in their home cage. On training days, mice typically received all their water in the task but were given additional water if required to maintain their body weight above 85% of their pre-restriction baseline weight.

Mice were trained on a sequence of reversal learning problems, each with the same structure but a different physical port layout. Each reversal learning problem used three nose-poke ports, out of the six or nine ports available in the operant box. One port was used for trial initiation; the other two were choice ports where reward could be obtained. During the initial training phase (Fig. 1a), ports not used in the current problem were covered. During recording sessions, ports used in all three problems presented in the session were exposed throughout, and unused ports were covered.

Each trial started with the initiation port lighting up, until the subject poked it, after which two choice ports both lit up. Mice chose one of the choice ports, which triggered a sound cue (250 ms long), indicating the trial outcome, with a pure tone (5 kHz) indicating that they will get a reward and white noise indicating reward omission. Reward was delivered at the termination of the auditory cue. A 2-second ITI started once the animal left the port after reward consumption or a non-rewarded choice. One in four randomly selected trials was a forced-choice trial, where a single randomly selected choice port lit up that the animals had to select. At any given point in time, one choice port had a high reward probability, and the other one had a low probability. Reward probability reversals were triggered 5–15 trials after the subject crossed a threshold of 75% correct choices (exponential moving average, tau = 8 trials).

In the initial training stage of experiment (Fig. 1), mice encountered a single problem (that is, port layout) per session and moved to the next problem the session after they had completed ten reversals on the current problem. In each problem, the first three reversals had reward probabilities of 0.9 and 0.1 at the good/bad choice ports. The fourth and fifth reversals had reward probabilities of 0.85 and 0.15, and the remaining reversals had reward probabilities of 0.8 and 0.2. In this phase, each session was 30 minutes long, and animals performed two sessions per day. The reward sizes during this stage were incrementally decreased from 15 µl in the beginning of the training to 4 µl, based on the animalsʼ performance. Each session started with a free reward given from each of the two choice ports. Mice were divided into three groups, with each group starting on a different layout. Sequentially presented layouts were chosen to be as different as possible, and the sequence of problem layouts was counterbalanced across animals.

Once mice had completed ten problems, we started presenting multiple problems in each session to prepare them for recording sessions where we sought to record neurons across multiple problems. Initially, mice were trained on two problems in a session in the nine port operant boxes subsequently used for recordings. Mice completed 12 different problems in this stage, with the port layout used in each chosen to be as different from the previous one as possible. The reward probabilities in this phase were always 0.8 and 0.2, and the reward size was 4 µl. After mice completed two reversal blocks on one layout, choice ports that were going to be a part of the new problem layout both lit up. Mice received a free reward from each of the new choice ports. Next, the new initiation port lit up, signaling mice where they could initiate a trial. See Supplementary Fig. 3 for all port layouts and counterbalancing used in the experiment.

### Behavior during recordings

During recordings, subjects completed four reversal blocks in each of three different problem layouts in every session. Task parameters were the same as during the two-layout-per-session training stage, with the exception that now subjects needed to complete four blocks on each problem before they were moved onto a new one. As before, the problem change was signaled by the two new choice ports lighting up until the subject collected a reward from each, followed by the new initiation port lighting up. Port layouts used during recording sessions were designed to allow us to ask specific questions of the neural activity and were all reflections of three basic layout types, each of which was presented once per session in a randomized order (results in Fig. 2b).

### Electrophysiological recordings and spike sorting

Cambridge NeuroTech 32 silicon channel probes were used for all recordings, with F series probes used for hippocampus and P series for mPFC. For hippocampal recordings, probes were implanted above the CA1 cell layer and lowered after surgery until they were in the layer, as assessed by local field potential and spike activity. For mPFC recordings, we lowered the probe ~100 µm on every recording day. Neural activity was acquired at 30 kHz with a 32-channel Intan RHD 2132 amplifier board (Intan Technologies) connected to an OpenEphys acquisition board. Behavioral, video and ephys data were synchronized using sync pulses output from the pyControl system. Recordings were spike sorted using Kilosort^66^ and manually curated using phy (https://github.com/kwikteam/phy). Clusters were classified as single units and retained for further analysis if they had a characteristic waveform shape, showed a clear refractory period in their autocorrelation and were stable over time.

### Surgery and histology

Subjects were taken off water restriction 48 hours before surgery and then anaesthetised with isoflurane (3% induction, 0.5–1% maintenance), treated with buprenorphine (0.1 mg kg^−1^) and meloxicam (5 mg kg^−1^) and placed in a stereotactic frame. A silicon probe mounted on a microdrive (Ronal Tool) was implanted into either mPFC (AP: 1.95, ML: 0.4, DV: −0.8) or dCA1 (AP: −2, ML: 1.7, DV: −0.7), and a ground screw was implanted above the cerebellum. Both DV coordinates are relative to the brain surface. Mice were given additional doses of meloxicam each day for 3 days after surgery and were monitored carefully for 7 days after surgery and then placed back on water restriction 24 hours before restarting task behavior. At the end of the experiment, electrolytic lesions were made under terminal pentobarbital anaesthesia to mark the probe location; animals were perfused; and the brains were fixed-sliced and imaged to identify probe locations.

### Data analysis

All analyses were carried out using custom Python code. Only sessions where animals completed three problems and four reversals in each problem were used for neural analyses.

### Time-in-trial alignment

Activity was aligned across trials by warping the time interval between trial initiation and choice to match the median interval across all recorded trials. Activity before trial initiation or after choice was not warped. Spike times that occurred between initiation and choice were converted into the aligned reference frame by linear interpolation between initiation and choice time. The firing rate of each neuron was calculated in the aligned reference frame at timepoints evenly spaced every 40 ms, from 1 second before trial initiation to 1 second after trial outcome, using a Gaussian kernel with 40-ms standard deviation. To compensate for the change in spike density due to time warping, spikes in the warped interval between initiation and choice were weighted by the stretch factor applied before evaluating the firing rate (Supplementary Fig. 2).

### Statistical significance

Significance of differences between brain areas in analyses reported throughout the paper were computed by shuffling the sessions of CA1 and PFC animals to obtain null distributions. To correct for multiple comparison across timepoints, the null distributions were formed by taking the peak difference between CA1 and PFC across timepoints in each permutation. This approach is a commonly used method for family-wise error correction for permutation tests^67^. Real differences in the data were compared against the 95th and 99th percentiles of such null distributions. All comparisons also survived a group test obtained by shuffling animal identities between regions (Extended Data Fig. 5). To establish the significance levels for the effects within regions (Figs. 4b and 6b), the firing rates were rolled with respect to trial identities, so that the autocorrelations between consequent trials were retained. Where parametric statistical tests were used, the data distribution was assumed to be normal, but this was not formally tested.

### Representational similarity regression analysis

We created representational similarity matrices that consisted of the Pearson correlation coefficients of neurons in 15 different conditions, defined by the trial stage, choice, outcome and problem number (Results and Fig. 4). Because neurons were not simultaneously recorded, we collapsed data across recording sessions for each brain region into a single matrix (cells × trial events) and then calculated the correlation matrix across cells between different trial events (that is, representational similarity). We used a linear regression to model the patterns of representation similarity in the data as a linear combination of RDMs:

$$r_{i,j} = \beta _0 + \mathop {\sum }\limits_{n = 1}^9 \beta _n{\mathrm{RDM}}_{n(i,j)} + \in _{i,j}$$

where r_(i,j)_ are elements of the RSA matrix, and *RDM*_n(i,j)_ are elements of the *n*th RDM. The set of RDMs used is shown in Fig. 4d. Before regressing the correlation matrices onto the RDMs, the diagonal elements from both were deleted, and a constant matrix of ones was added to the design matrix to account for any condition-independent correlation between neurons. We plotted the CPDs from the regression model described above. The CPD was defined as:

$${\mathrm{CPD}}\left( {{\mathrm{RDM}}_i} \right) = \left( {{\mathrm{SSE}}_{\sim i} - {\mathrm{SSE}}_{{\mathrm{full}}\,{\mathrm{model}}}} \right)/{\mathrm{SSE}}_{\sim i}$$

where SSE_*∼i*_ refers to the sum of squares from a regression model excluding the RDM_*i*_ of interest, and SSE_full model_ is the sum of squares from a regression model including all the RDMs. CPDs describe how much unique variance each RDM accounts for in the RSA matrix calculated from firing rates.

### Decoding analyses

We trained a support vector classifier (implemented using sklearn.svm.SVC) to classify stages of the trial (Initiation, A choice, B choice, A reward, B reward, A no-reward and B no-reward) from neural activity on one problem and tested how it performed on a different problem. This was computed for all problem pairs, and the mean decoding accuracy for each trial stage was shown in a form of a confusion matrix (Fig. 4f).

We then analyzed these confusion matrices to look for patterns of decoding associated with a representation of (1) physical port, (2) trial stage (initiation, choice and type of outcome) and (3) abstract choice. In one of our problem layout pairs, initiation port became a B choice (layout 2 to layout 3), and, in another, initiation became a B choice (layout 3 to layout 2), so mistakes made by the decoder between B choice and Initiation in these pairs indicate a prominent representation of port location. Decoding errors between A choices and B choices, A rewards and B rewards and A no-rewards and B no-rewards indicate a representation of trial stage. Lastly, representation of an abstract choice (A vs B) independent of port location was computed by summing cells corresponding to the same abstract choice but in a different physical location across problems. Statistical significance of differences between PFC and CA1 in decoding patterns was established by permuting animal identities between regions and comparing the real differences against the 95% confidence interval of the shuffle.

### Surprise measure

To investigate the time course of how quickly the firing rates of neurons change in response to layout changes (Extended Data Fig. 8), we used the ‘surprise’ measure from the information theory:

$$s(x_{ij}) = \left( {\frac{1}{n}\mathop {\sum }\limits_{i = 1}^n x_{ij} - \mu _{kl}} \right)^2/\sigma _{kl}^2$$

where x_ij_ is the firing rate of one neuron on a given trial *i* and problem layout *j*; and *μ*_k_ and *σ*_k_ are the baseline mean and standard deviation of the firing rate of that neuron on a particular problem layout. If *j* *=* *k*, then the s(x_ij_) on each trial *i* is calculated based on the mean firing rate *μ* and standard deviation *σ* of the withheld trials from the same problem. More precisely, to calculate how much the firing rates change during the same problem layout, s(x_ij_) was calculated on the ten trials before the problem layout switch (‘test’ within problem), where *μ*_k_ and *σ*_k_ were calculated on the ten trials before those ‘test’ trials (‘train’ within problem). If *j* ≠ *k*, then the s(x_ij_) on each trial *i* was calculated based on the mean firing rate *μ* and standard deviation *σ* of the withheld trials from a different problem. So, to estimate how much the firing rates change after the problem layout switch, s(x_ij_) was calculated on the 20 trials after the problem layout switch (‘test’ between problems), where *μ*_k_ and *σ*_k_ were calculated from the ‘train’ trials from a different layout. This measure was calculated for each neuron separately and then averaged across all neurons for each brain region.

### SVD

SVD was performed using the numpy linalg.svd function in Python. SVD is a principal component analysis technique that decomposes any n × m matrix into a product of three matrices:

$$D = U{\Sigma}V^T$$

where *D* comprises the data matrix to be decomposed; *U* and *V*^*T*^ are sets of singular vectors capturing patterns of covariation in the data; and Σ is a diagonal weight matrix.

In our SVD analyses, each row of *D* was the demeaned, trial-aligned activity of one neuron across each timepoint of four concatenated trial types: rewarded A choices, non-rewarded A, rewarded B and non-rewarded B. So the shape of D was [n_neurons, 4 × n_timepoints_per_trial]. The columns of *U* are vectors that we term cellular modes because each is a set of weights over neurons, representing groups of neurons whose activity covaries. Each cellular mode has a corresponding row in *V* ^*T*^ that we term a temporal mode, as it is a set of weights over timepoints, representing the time course of the cellular mode’s activity. Each temporal mode spans the same set of timepoints as the data matrix and, hence, captures variation both over time-in-trial and trial-type. As both modes are unit vectors, their contribution to the total data variance is determined by the corresponding element of the diagonal matrix Σ.

The cellular modes are given by eigendecomposition of the covariances between neurons, as can be seen from the following:

$$DD^T = (U{\Sigma}V^T)(U{\Sigma}V^T)^T$$

$$DD^T = (U{\Sigma}V^T)(V{\Sigma}U^T)$$

$$DD^T = U{\Sigma}^2U^T$$

As *DD*^*T*^ is the non-normalized covariance between neurons across timepoints, Σ^2^ is a diagonal matrix of eigenvalues, *U* are the corresponding eigenvectors and *U*^*T*^ = *U*^−1^ because *U* is an orthonormal basis.

Similarly, the temporal modes are given by eigendecomposition of the covariances between timepoints:

$$D^TD = \left( {U{\Sigma}V^T} \right)^T(U{\Sigma}V^T)$$

$$D^TD = (V{\Sigma}U^T)(U{\Sigma}V^T)$$

$$D^TD = V{\Sigma}^2V^T$$

As *D*^*T*^*D*^*T*^ is the non-normalized covariance between timepoints across neurons, Σ^2^ is a diagonal matrix of eigenvalues, *V* are the corresponding eigenvectors and *V*^*T*^ = *V*^−1^ because *V* is an orthonormal basis.

Our goal was to test whether cellular and temporal patterns generalize across different problems by quantifying how well cellular and/or temporal modes from one problem explained variance in another. As a control for drift in representations over time, we compared generalization between problems with generalization to held-out data from the same problem. To do this, we constructed separate data matrices for the first and second half of each problem:

$$D_{i,h} = U_{i,h}{\Sigma}_{i,h}V_{i,h}^T$$

where *i* is the problem number $i = \{ 1,2,3\}$, and *h* is the half of the problem that the data are taken from $h = \{ f,s\}$. We can then compare generalization between the second half of one problem with the first half of the next with generalization between first and second half of the same problem, to ensure that any drift is matched between within-problem and cross-problem comparisons.

We quantified three different ways in which activity patterns might generalize between problems. (1) Generalization of temporal modes irrespective of whether they recruited the same neurons. This corresponds to the same trial events being represented but not necessarily by the same neurons. (2) Generalization of cellular modes irrespective of whether they have the same time course. This corresponds to the same cell assemblies co-activating but not necessarily representing the same trial events. (3) Generalization of cellular and temporal mode pairs. This corresponds to the same cell assemblies representing the same trial events across problems.

To quantify how well temporal modes generalized across problems, we projected the data matrix from half of one problem on the temporal modes from an adjacent half of a different problem:

$$M_v^{\mathrm{cross}} = D_{2,f}V_{1,s}$$

The total variance explained by each temporal mode for this problem pair is given by squaring the elements of $M_v^{\mathrm{cross}}$ and summing over neurons. We average across all adjacent problem pairs and plot the cumulative variance explained as a function of the number of temporal modes used.

The corresponding within-problem variance explained is given by projecting the data matrix from half of one problem onto the temporal modes from the other half of the same problem:

$$M_v^{\mathrm{same}} = D_{1,f}V_{1,s}$$

Similarly for the cellular modes, the cross-problem generalization was given by projecting the data matrix from half of one task on the cellular modes from an adjacent half of a different problem:

$$M_U^{\mathrm{cross}} = U_{1,s}^TD_{2,f}$$

The total variance explained by each cellular mode for this problem pair is given by squaring the elements of $M_U^{cross}$ and summing over timepoints. Again, we average across all adjacent problem pairs and plot the cumulative variance explained as a function of the number of cellular modes used.

The corresponding within-problem variance explained is given by projecting the data matrix from half of one problem onto the cellular modes from the other half of the same problem:

$$M_U^{\mathrm{same}} = U_{1,s}^TD_{1,f}$$

To quantify how well pairs of neural and temporal patterns generalized between problems, we projected the data matrix from half of one problem on the cellular and temporal modes from an adjacent half of a different problem:

$${\Sigma}_{\mathrm{cross}} = U_{1,s}^TD_{2,f}V_{1,s}$$

Σ_cross_ is not diagonal; however, if the same cell assemblies perform the same roles in two problems, the temporal and cellular modes will align, and Σ_cross_ will have high weights on the diagonal. We, therefore, plotted the cumulative sum of the squared weights of the diagonal elements. Because we had different numbers of neurons in each brain region, Σ_cross_ was normalized by the number of neurons recorded from the respective brain region.

The corresponding within-problem variance explained is given by projecting the data matrix from half of one problem onto the cellular and temporal modes from the other half of the same problem:

$${\Sigma}_{\mathrm{same}} = U_{1,s}^TD_{1,f}V_{1,s}$$

To determine the significance of the differences between two regions, we compared differences in the data between PFC and CA1 against a null distribution of differences between areas under the curve by shuffling the sessions between CA1 and PFC animals.

### Estimating policy

We obtained a trial-by-trial estimate of subjectsʼ behavioral policy using a logistic regression predicting current trial choice, using the history of choices, rewards and choice × reward interactions (Fig. 6a). This gave an estimate on each trial of the probability that the animal would choose A, which we term the animalʼs policy. We used this policy and its interaction with current choice (policy × choice), together with current trial events (choice, outcome and outcome × choice interaction), to predict neural activity in a linear regression, quantifying the variance explained by each predictor at each timepoint as the CPD (Fig. 6b).

To understand whether policy representations generalized between problems, we conducted this linear regression separately for A and B choices (dropping the current trial choice predictor from the regression), obtaining one vector of coefficients for A choices and one for B choices, indicating how policy affected the activity of each neuron, which we term policy representations. As the policy representation may change across the trial, we did this for a set of time windows across the trial, obtaining a policy representation for each timepoint for A and B choices. We quantified how similar policy representations were between problems and timepoints as the Pearson correlation, to obtain the matrices shown in Fig. 6c. Finally, to understand whether these across trial policy signals might also be tied to representations of unique trial stages, we examined time slices through the correlation matrices at initiation, choice and outcome times. The differences between these signals at each timepoint were then compared against null distributions described in the ‘Statistical significance’ section.

### Additional controls for physical movement

To provide additional controls for movement-related activity, we sought to eliminate the effect of an animal’s position, velocity and acceleration on firing rates before performing all our subsequent analyses. To do this, we used DeepLabCut^68^ pose estimation software to extract the animal’s nose position in each session. Because the cameras in the operant boxes were located above the animal, there were some artifacts in the tracking caused by the occlusion of the nose by the ports and the head cap, which caused the estimated position to jump to incorrect locations. To correct for this, we first removed all samples where the likelihood of correct estimation output by DeepLabCut was below 90%. We then removed samples adjacent to jumps in position larger than ten times the standard deviation of displacements between frames, estimated using the 16th and 84th percentiles of the displacement distribution. We then removed samples that were not in contiguous groups of at least five. After this artifact removal step, we interpolated the missing data, taking advantage of the fact that the movements of the ears and nose are highly correlated, such that the trajectories of the ears provide information about movements of the nose when the nose is occluded. The interpolation was implemented by minimizing a cost function with two terms: (1) the sum of squared derivatives of the nose position, which promotes linear interpolation of missing data, and (2) the sum of squared differences between the derivatives of the ear and nose positions, which promotes the interpolated trajectory of the nose tracking those of the ears.

Next, because we had the ground truth of our port locations in physical space, we performed a linear registration and transformed the 2D coordinates extracted from the video from the oblique camera view to a more informative horizontal view of the wall of the ports. Finally, we used our behavioral data to find when the animals were inside the ports and corrected for any inaccuracy in our DeepLabCut data by placing these coordinates inside the ports.

As we did not expect that the 2D coordinates of animal’s nose position would be linearly related to neural firing rates (for example, due to previously reported existence of ‘place cells’ in CA1), we first needed to create vectorized ‘occupancy maps’ (Extended Data Fig. 7a). Specifically, we defined a set of Gaussian ‘radial basis functions’ with the centers randomly selected from an animal’s 2D coordinates in each session and a standard deviation of 1 cm. Next, for each timepoint, we calculated the activity of each basis function (Gaussian in distance from center of this field), resulting in a matrix of shape [time, n_basis_functions].

To account for cross-correlations in this matrix, we next performed a principal component analysis to extract the first ten orthogonal occupancy components across time accounting for >95% of variance. To confirm that our key results could not be explained by movement-related parameters, we repeated our main analyses using the residual firing rates from a linear model predicting the firing of each neuron using these occupancy components, as well as the acceleration and velocity of the animal at each timepoint (Extended Data Fig. 7a,b). Because we did not have video data for all our animals due to technical limitations at the time of experiments, the significance between brain areas in these analyses was only computed by shuffling the sessions of CA1 and PFC animals to obtain null distributions and correcting for multiple comparisons as before (see the ‘Statistical significance’ section).

### Reporting summary

Further information on research design is available in the Nature Research Reporting Summary linked to this article.

## Online content

Any methods, additional references, Nature Research reporting summaries, source data, extended data, supplementary information, acknowledgements, peer review information; details of author contributions and competing interests; and statements of data and code availability are available at 10.1038/s41593-022-01149-8.

## Supplementary information

Supplementary Information Supplementary Figs. 1–3

Reporting Summary

## Data Availability

Data from the study are available to download at 10.6084/m9.figshare.19773334.

## Code availability

Jupyter Notebooks for generating the figures in the study are available at https://github.com/veronikasamborska1994/notebooks_paper.
