# Supplementary material for: Complementary task representations in hippocampus and prefrontal cortex for generalizing the structure of problems
Source: Nat Neurosci. 2022 Sep 28;25(10):1314–26. doi: 10.1038/s41593-022-01149-8 (PMC9534768; doi:10.1038/s41593-022-01149-8)
Supplement: Supplementary file 1 — Supplementary Figs. 1–3 [file 41593_2022_1149_MOESM1_ESM.pdf]

nature portfolio

https://doi.org/10.1038/s41593-022-01149-8

Supplementary information

Complementary task representations in hippocampus and prefrontal cortex for generalizing the structure of problems

In the format provided by the authors and unedited

Supplementary Figures

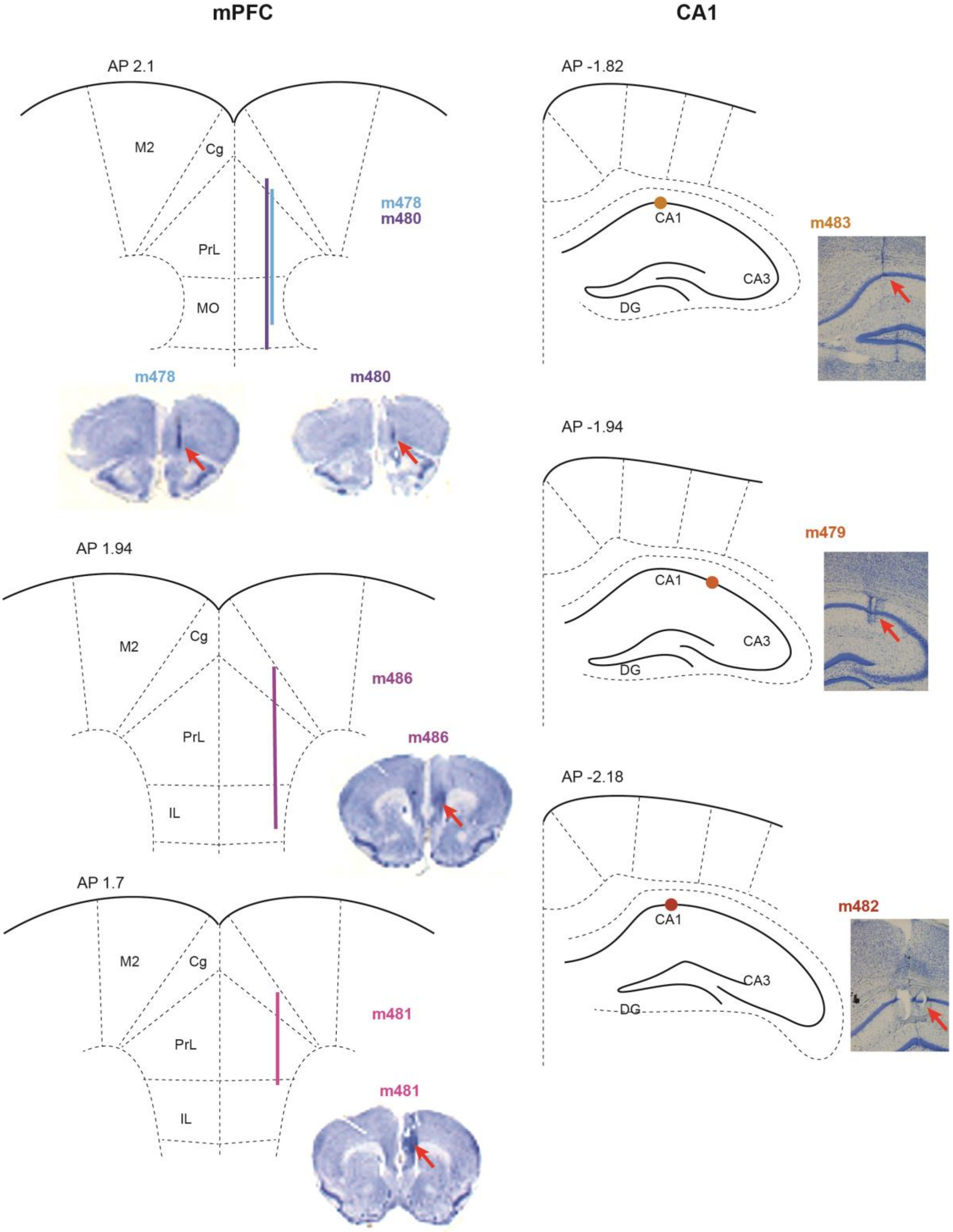

Supplementary Figure: 1. Location of Recording Sites. mPFC probes were advanced on each session whereas CA1 probes were static throughout recordings (for more details see *Electrophysiological Recordings and Spike Sorting Methods*). mPFC recordings were mostly from prelimbic area but also in some cases reached infralimbic area, medial orbital and cingulate**.**

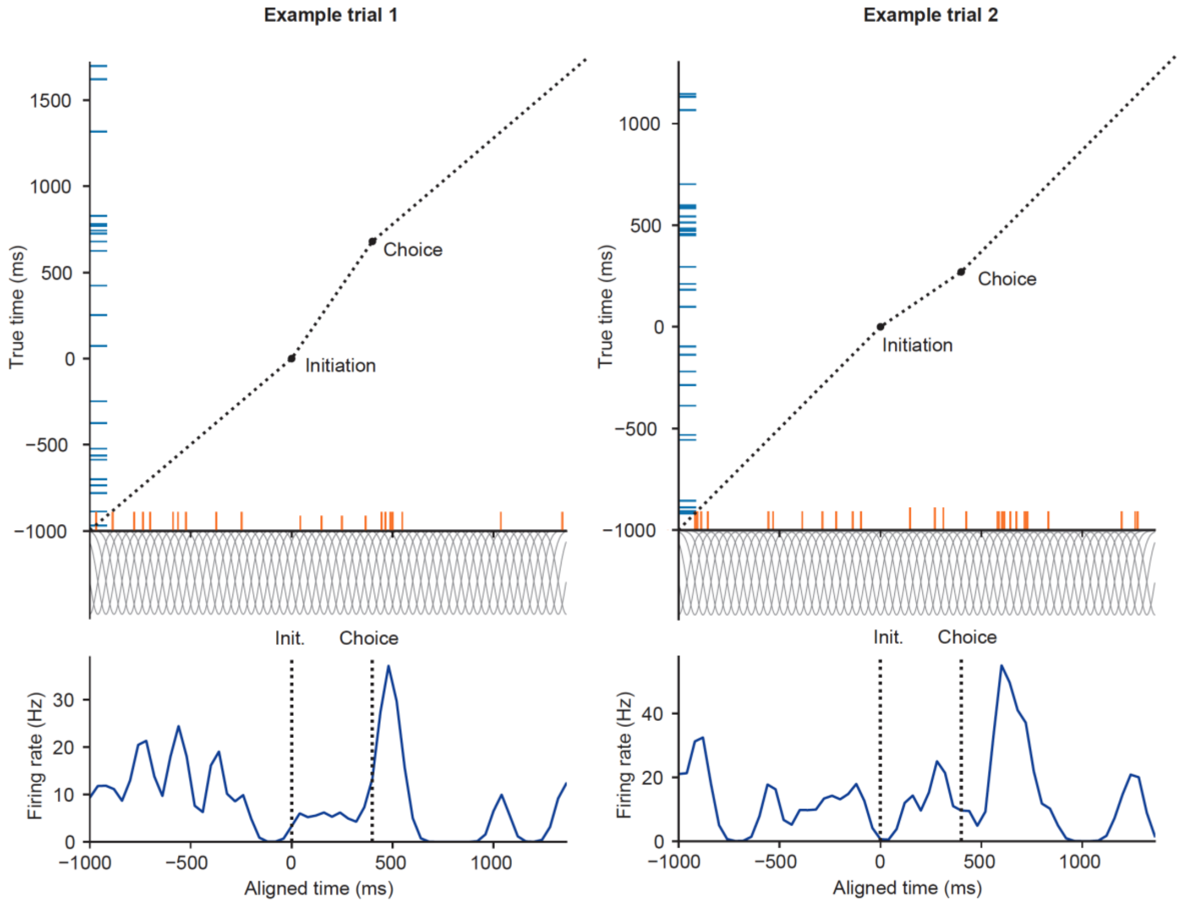

Supplementary Figure: 2. Trial alignment of activity. Diagram illustrating alignment of spike activity across trials. Trials were aligned using the times of initiation and choice port entry, by warping the interval between these two events to match the median interval. Top panels show spike times in the true (blue ticks) and aligned (orange ticks) time reference frames. Spike times were transformed by linear interpolation between the reference points. The output firing rate (bottom panels) was calculated at points spaced every 40ms in the aligned reference frame using Gaussian smoothing (40ms standard deviation) of the spike train. To compensate for the change in spike density due to the time warping, spikes were weighed by the stretch factor between the true and aligned reference frames (weighting is indicated by height of the orange ticks) prior to Gaussian smoothing.

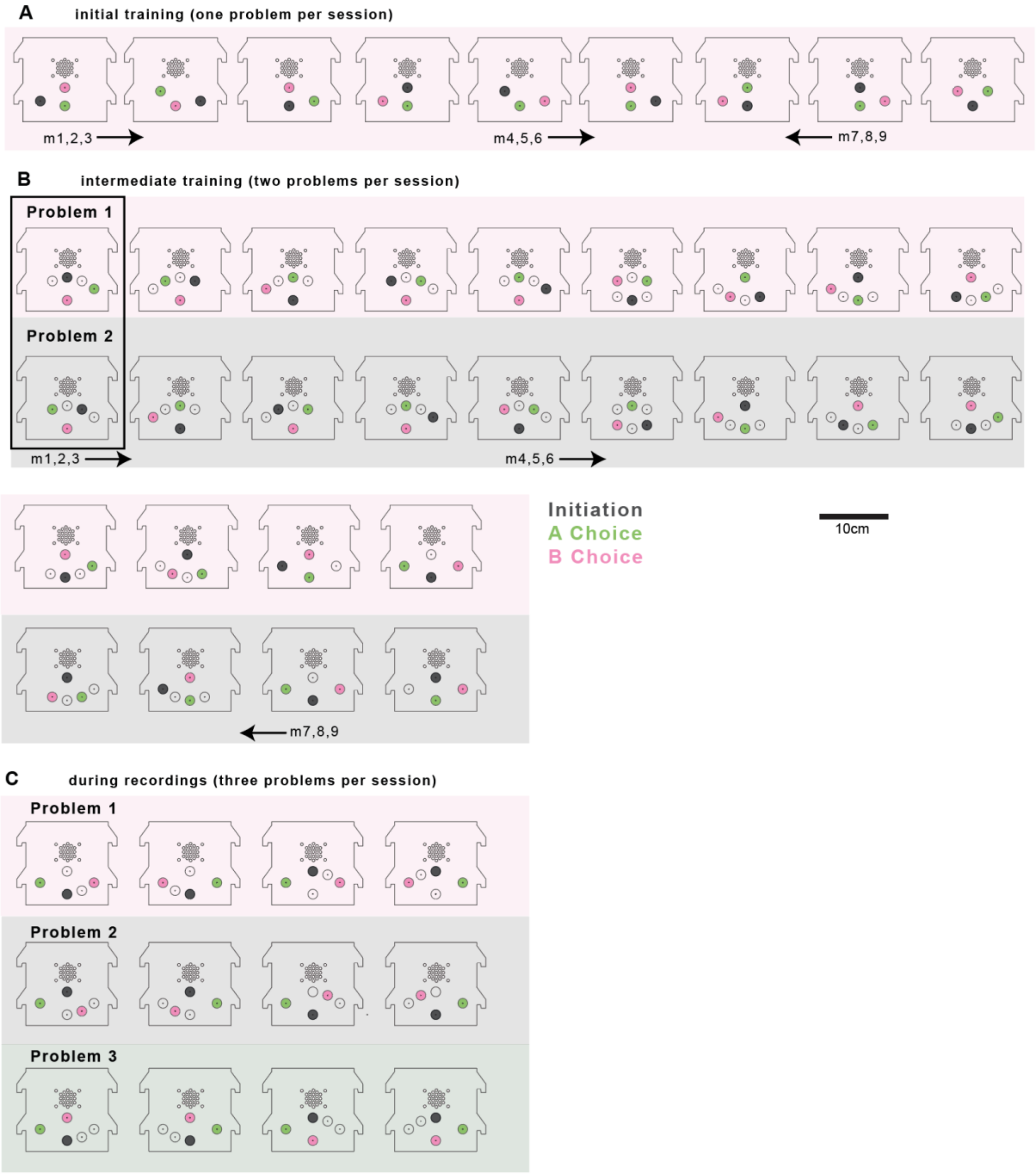

Supplementary Figure: 3. Counterbalancing and port layouts used throughout the experiments. Port layouts used for initial training (A), intermediate training (B) and recording sessions (C), showing the locations of the initiation (grey), A choice (green) and B choice (pink ports) on the wall of the box. Ports that were not used in any of the problems presented in a session were covered up and are not shown on the diagrams. Ports that were exposed but not used in a given problem are shown in white. In B and C the set of problems used in a single session are arranged vertically. During initial and intermediate training stages, the presentation order of different configurations was counterbalanced across animals by randomly assigning mice to 3 in groups each of 3 animals. The starting configurations for the different animals are indicated under the layouts, with an arrow indicating the direction the group subsequently progressed through the layouts across training. The layouts shown for the recordings are labelled 1,2 and 3 for consistency with how they are described in the results text, but the actual

presentation order within each session was randomised. For more details on the training protocol see *Behavioural Training Methods*.
